# Sex and age differences in social and cognitive function in offspring exposed to late gestational hypoxia

**DOI:** 10.1186/s13293-023-00557-0

**Published:** 2023-11-11

**Authors:** Steve Mabry, E. Nicole Wilson, Jessica L. Bradshaw, Jennifer J. Gardner, Oluwadarasimi Fadeyibi, Edward Vera, Oluwatobiloba Osikoya, Spencer C. Cushen, Dimitrios Karamichos, Styliani Goulopoulou, Rebecca L. Cunningham

**Affiliations:** 1 Department of Pharmaceutical Sciences, School of Pharmacy, University of North Texas Health Science Center, 3500 Camp Bowie Boulevard, Fort Worth, TX 76107 USA; 2 Department of Physiology and Anatomy, University of North Texas Health Science Center, Fort Worth, TX 76107 USA; 3 Texas College of Osteopathic Medicine, University of North Texas Health Science Center, Fort Worth, TX 76107 USA; 4 North Texas Eye Research Institute, University of North Texas Health Science Center, 3430 Camp Bowie Blvd, Fort Worth, TX 76107 USA; 5 Department of Pharmacology and Neuroscience, University of North Texas Health Science, Fort Worth, TX 76107 USA; 6 Departments of Basic Sciences, Gynecology and Obstetrics, Lawrence D. Longo, MD Center for Perinatal Biology, Loma Linda University School of Medicine, Loma Linda, CA 92350 USA

**Keywords:** Prenatal programming, Sex differences, Chronic intermittent hypoxia, Marble burying behaviors, Open field, Social behaviors, Morris water maze, Hippocampus

## Abstract

**Background:**

Gestational sleep apnea is a hypoxic sleep disorder that affects 8–26% of pregnancies and increases the risk for central nervous system dysfunction in offspring. Specifically, there are sex differences in the sensitivity of the fetal hippocampus to hypoxic insults, and hippocampal impairments are associated with social dysfunction, repetitive behaviors, anxiety, and cognitive impairment. Yet, it is unclear whether gestational sleep apnea impacts these hippocampal-associated functions and if sex and age modify these effects. To examine the relationship between gestational sleep apnea and hippocampal-associated behaviors, we used chronic intermittent hypoxia (CIH) to model late gestational sleep apnea in pregnant rats. We hypothesized that late gestational CIH would produce sex- and age-specific social, anxiety-like, repetitive, and cognitive impairments in offspring.

**Methods:**

Timed pregnant Long-Evans rats were exposed to CIH or room air normoxia from GD 15–19. Behavioral testing of offspring occurred during either puberty or young adulthood. To examine gestational hypoxia-induced behavioral phenotypes, we quantified hippocampal-associated behaviors (social function, repetitive behaviors, anxiety-like behaviors, and spatial memory and learning), hippocampal neuronal activity (glutamatergic NMDA receptors, dopamine transporter, monoamine oxidase-A, early growth response protein 1, and doublecortin), and circulating hormones in offspring.

**Results:**

Late gestational CIH induced sex- and age-specific differences in social, repetitive, and memory functions in offspring. In female pubertal offspring, CIH impaired social function, increased repetitive behaviors, and elevated circulating corticosterone levels but did not impact memory. In contrast, CIH transiently induced spatial memory dysfunction in pubertal male offspring but did not impact social or repetitive functions. Long-term effects of gestational CIH on social behaviors were only observed in female offspring, wherein CIH induced social disengagement and suppression of circulating corticosterone levels in young adulthood. No effects of gestational CIH were observed in anxiety-like behaviors, hippocampal neuronal activity, or circulating testosterone and estradiol levels, regardless of sex or age of offspring.

**Conclusions:**

Our results indicate that hypoxia-associated pregnancy complications during late gestation can increase the risk for behavioral and physiological outcomes in offspring, such as social dysfunction, repetitive behaviors, and cognitive impairment, that are dependent on sex and age.

**Supplementary Information:**

The online version contains supplementary material available at 10.1186/s13293-023-00557-0.

## Plain Language Summary

Sleep apnea during late pregnancy is a common pregnancy complication that can impact the brain development of children born to mothers with sleep apnea. Children with impaired brain development may present with decreased social skills, memory issues, anxiety, and compulsivity. It is unclear if there is a cause and effect relationship between sleep apnea during late pregnancy and behavioral changes in offspring. Additionally, it is unknown whether male or female sex or age of the offspring affects these relationships. In this study, we exposed pregnant rats to a model of sleep apnea called chronic intermittent hypoxia (CIH) within late gestation and examined the behavior of the offspring and brain activity during puberty and young adulthood. We found that CIH during late pregnancy had long-term effects in the offspring that were different in males and females. Notably, female offspring displayed social impairments in response to late gestation CIH, whereas male offspring displayed cognitive dysfunction.

**Supplementary Information:**

The online version contains supplementary material available at 10.1186/s13293-023-00557-0.

## Highlights

- This is the first study to examine the effects of late gestational CIH on social, anxiety, and cognitive function in rat offspring.
- Short term hypoxia during late gestation induced sex- and age-specific differences in offspring social and cognitive function.
- Late gestational hypoxia induced sustained social impairments and corticosterone dysregulation in female offspring that were present during puberty and young adulthood.

**Supplementary Information:**

The online version contains supplementary material available at 10.1186/s13293-023-00557-0.

## Background

Hypoxia is a main characteristic of gestational sleep apnea and a culprit for other obstetric complications, such as gestational diabetes and preeclampsia [1–4]. Gestational sleep apnea affects 8–26% of pregnancies [5–8], and is associated with impaired fetal development underlying fetal central nervous system (CNS) dysfunction, growth restriction, and organ damage [9–13]. Sex plays a role in risk from gestational complications, such as gestational sleep apnea [10, 11]. In pregnancies that experience complications, there is a sex dependent risk for neonatal fatality and morbidities (e.g., survival rates, growth restriction, metabolic dysfunctions) [14–17]. In addition to adverse perinatal outcomes, gestational hypoxia can increase the risk for chronic disorders, such as neurodevelopmental disorders in offspring later in life [18, 19].

Experimental rodent models of gestational hypoxia have identified multiple hypoxia-sensitive behavioral indices in offspring, such as gestational hypoxia-induced impairments in social function, cognitive function, anxiety-like and repetitive behaviors [20–23]. Sex is an important factor in the vulnerability of offspring to gestational hypoxia, in which adult male offspring have greater social, cognitive, and anxiety-like dysfunctions than adult female offspring [22–24], but there is a paucity of studies on the pubertal consequences of gestational hypoxia. Puberty is a critical period for social, endocrine, and cognitive maturations [25, 26]. It is important to note that many studies examining gestational hypoxia utilized sustained hypoxic exposure for either greater than 15 gestational days or a single exposure lasting between two to eight hours on one gestational day [21–24], rather than a chronic intermittent hypoxia (CIH) exposure that models the fragmented hypoxic episodes present in sleep apnea [9]. Notably, a recent study demonstrated CIH exposure for 12 days during mid to late gestation impaired memory and social functions during puberty and adulthood in male rat offspring but had no impact on female offspring [20]. Nonetheless, incorporating a shorter CIH exposure that is limited to late gestation would model the majority of sleep apnea cases that present in the last trimester of human pregnancy [6, 8]. It is unknown whether a short exposure to chronic intermittent hypoxia during late gestation would impair social, anxiety-like, or cognitive function.

Social, anxiety-like, repetitive and cognitive impairments are associated with hippocampal dysfunction [27–30]. The hippocampus is sensitive to hypoxic insults that can result in behavioral deficits [31–33]. Hippocampal-mediated dysfunctions have been linked with age-related changes in neurotransmitters and circulating hormones. Prior studies have found hippocampal dysfunction associated with differences in glutamatergic, dopaminergic, and serotonergic pathways [34–36]. Further, hippocampal function is sensitive to steroid hormone levels, with impairments observed following dysregulation of testosterone [31, 37], estradiol [38, 39], and corticosterone [37, 40].

Since gestational hypoxia can have a long-lasting impact on offspring physiology, brain function, and behavior, it is important to understand the influence of sex and age on late gestational hypoxia-associated chronic outcomes. To examine these relationships, we used a CIH protocol during gestational days 15–19 in rats to model late gestational sleep apnea [9]. We chose this model of gestational CIH as the incidence of gestational sleep apnea is higher during late gestation [6, 8], during which time fetal CNS development is occurring [41, 42]. Using this CIH protocol, we previously examined ultrasonic vocalizations (USVs) and open field locomotor activity in offspring at different ages (puberty and young adulthood) to examine the impact of late CIH exposure during gestational days 15–19 on fetal neurodevelopment [9]. We found that gestational CIH had no impact on motor function (distance traveled or rearing) or stress-associated 22 kHz USVs, but CIH did alter positive affect-associated 50 kHz USVs in male and female offspring [9]. Importantly, USVs are dependent on multiple maturation domains (e.g., motor function, social function, and anxiety-like behavior [43–47]), indicating that gestational CIH could impact multiple maturation domains. Therefore, in addition to our previous studies examining USVs and locomotor activity, we examined the effects of gestational CIH on social, cognitive, repetitive, and anxiety-like behaviors displayed by offspring. Within the same cohort of offspring, we present here offspring behaviors that include center arena behaviors, marble burying, social function, and spatial memory. We hypothesized that late gestational CIH would produce sex- and age-specific social, cognitive, repetitive, and anxiety-like impairments in offspring.

## Methods

### Animals

This study was part of a larger study that examined the impact of late-stage gestational hypoxia on maternal and offspring physiology [9]. All experiments were conducted using timed pregnant Long-Evans rats (aged 8–10 weeks, Charles River, Wilmington, MA). Dams arrived at animal facilities on gestational day (GD) 5–7, in which the sperm plug was observed on GD0. Dams were single housed in a 12 h:12 h light/dark cycle with lights on at 0900 h and were provided food and water ad libitum. Dams were allowed to habituate for 4 days prior to the initiation of the chronic intermittent hypoxia (CIH) protocol that was performed during late gestation (GD 15–19) prior to delivery (GD 22–23). Maternal and fetal biometric analyses are reported in our prior publication [9]. Following delivery, the sex of the offspring was determined via anogenital distance and confirmed prior to weaning. Litters were reduced to 8 pups/litter and, when possible, to equal number of males and females. Weaning occurred on postnatal day (PND) 28. Approximately 4 male and 4 female pups in each litter were randomly assigned to either pubertal (PND 40–45) or young adult (PND 60–65) offspring experimental groups. Based on gestational exposure to hypoxia or normoxia (room air), offspring were included in the following treatment groups. Puberty: Male Normoxic (n = 12), Male CIH (n = 16), Female Normoxic (n = 10), Female CIH (n = 11); Young adult: Male Normoxic, (n = 13) Male CIH (n = 13), Female Normoxic (n = 10), Female CIH (n = 9).

Pubertal and young adult offspring were housed with 1–2 littermates of the same sex to accommodate for uneven distribution of male or female offspring. Male and female offspring were housed in separate rooms in our animal facility. Offspring were housed in rooms on a 12 h:12 h reverse light/dark cycle where lights were off at 0900 h. Reverse lighting allowed behavioral testing to be conducted during the offspring’s active phase of the circadian cycle. Food and water were provided ad libitum for all rats*.* To acclimatize the offspring to operator handling and reduce stress responses during behavior testing, offspring were handled daily, beginning approximately 10 days prior to the start of behavior testing. At the conclusion of behavior testing, the offspring were anesthetized with 2–3% isoflurane and euthanized via decapitation during the active phase of the circadian cycle between 0900 and 1100 h on PND 48 (puberty) or PND 66 (young adult). All experiments were conducted in agreement with the Guide for the Care and Use of Laboratory Animals of the National Institutes of Health and the ARRIVE guidelines. These protocols were approved by the Institutional Animal Care and Use Committee of the University of North Texas Health Science Center.

### Chronic intermittent hypoxia protocol

Timed pregnant female rats were assigned to receive CIH (n = 8) or room air normoxic (NORM; n = 8) treatments for 8 h starting at 0900 h during their sleep phase of the circadian cycle on GD 15–19. Four days prior to the initiation of the CIH protocol, the home cages (clear plastic containers with sufficient bedding and nesting materials for the dams) of the timed pregnant females were placed into Oxycycler chambers (76.2 × 50.8 × 50.8 cm, BioSpherix, Lacona, NY, USA) to acclimatize the dams to the chambers under NORM conditions. The CIH protocol consists of oxygen reduction from 21% (room air) to 10% oxygen, then returned to 21% oxygen in 6-min cycles with 10 cycles/hour over 8 h/day during the sleep phase of the rat circadian cycle (lights on) for a period of 5 days, as previously described [9]. Following the end of the CIH protocol (GD 19), the cages containing the dams were left in the Oxycycler chambers with the chambers open to room air, and the dams were not disturbed until 24 h following delivery for sexing of the pups.

### Behavioral tasks

Behavioral studies were conducted either during puberty (PND 40–45) or young adulthood (PND 60–65) over the course of 1 week during the active phase of their circadian cycles, from 0945 to 1700 h. This cross-sectional design, in which rats were only tested either during puberty or young adulthood, was performed to prevent learning due to prior experience with these tasks (i.e., test battery effects). Notably, test battery effects have been reported in open field tests [48–50] and Morris water maze [48, 49], which are behavior tests used in this study. Therefore, to avoid this confound, one behavioral task per index of interest was performed and rats were assessed at either puberty or young adulthood. The order of the behavior tests was randomized. Male offspring were tested at least 1 h before female offspring to avoid potential confounding effects of pheromones on behavior. All testing equipment (e.g., marbles, arenas, tanks) were thoroughly cleaned with 70% ethanol between each rat. All behavior studies were conducted under red lighting and recorded for later analysis by an investigator blinded to treatment groups. Behavior tests were used to assess hippocampal-dependent behaviors, such as repetitive behaviors (marble burying test) [51, 52], social function (social behaviors, social disengagement, social withdrawal, aggression) [27, 53, 54], anxiety-like behaviors (center entries and center duration in an open field, exploration) [27, 29], and spatial learning and memory (Morris water maze) [27, 51]. Methods and results for USVs quantified from this test are reported in Wilson et al. [9].

### Marble burying test – repetitive behaviors

This test was used to observe repetitive behaviors [55, 56]. To conduct this test, the floor of the testing arena (50 × 25x30 cm) was thoroughly covered with 1 cm of rodent bedding litter to allow the rats to easily bury marbles. Twenty marbles of similar color and size (1.5 cm) were positioned in a 4 × 5 grid spaced evenly on one side of the arena base, and a pre-test photograph was taken. At the beginning of the test, a single rat was placed on the opposite side of the arena facing away from the marbles (Additional file 1: Fig. S1A). Each rat was given 10 min to explore and interact with the marbles. Operators visually monitored the experiment and manually recorded behaviors. After 10 min, the rat was removed and the arena was photographed for later quantification of the number of buried marbles. A marble was considered buried if 75% or more of the marble was covered with bedding litter. Post hoc quantification was performed by an experimenter blinded to the treatment of the rats by examining the photographs taken before and after the test.

### Social function

To quantify social behaviors, social disengagement, and social withdrawal in a group social setting, rats were placed into a novel arena (50 × 25 × 30 cm) with novel conspecific rats of the same sex for 10 min. Due to the uneven numbers in some of the study groups and to maintain the novelty of the arena, social group settings were either 2 or 3 rats of the same sex. We observed no differences in behaviors due to these social group numbers (Additional file 9: Table S1). The arena was lined with litter bedding (1.5 cm). To allow the rats to retreat from the novel arena, a foil covered semi-cylindrical tube (6.5 × 9 × 20.5 cm) was placed into the arena. Behaviors assessed included: (1) social behaviors (sniffing + following + climbing_under_ + climbing_over_ = social behavior composite score) [22, 57], (2) social disengagement behavior (percentage of nose-to-nose engagements where the rat was the first to disengage from interaction with a conspecific; Additional file 1: Fig. S1B), (3) social withdrawal (rats that did not perform nose-to-nose interactions with conspecifics), (4) total exploratory behaviors (rearing + digging + tube_climb_ + tube_sniff_ + tube_chew_) [58, 59], (5) aggressive behaviors (kicking + biting + dominance posture + boxing + fighting = aggressive behavior composite score) [54, 60–62]. Due to technical issues with video recordings during the social behavior testing, footage for 9 young adult females (normoxic n = 4; CIH n = 5) was lost and not available for analysis for social disengagement behavior, social withdrawal, and exploratory behavior.

### Open field center—anxiety-like behavior

Anxiety-like behaviors were assessed using a novel open field arena (40.64 × 40.64 × 38.1 cm) with the bi-directional main field bar (San Diego Instruments Photobeam Activity System (PAS)-Open field arena). Rats were allowed 10 min in the novel open field arena to explore. Anxiety-like behaviors can be assessed by the time spent (duration) or entries into the center of the open field [63]. To quantify these behaviors, a center zone was created within the PAS system to denote the number of entries (frequency) to the zone and time spent (duration) within the zone versus time spent around the edge [31]. Locomotor behaviors quantified from this test are reported in Wilson et al. [9].

### Morris water maze—spatial memory

To examine spatial memory and learning, the Morris water maze test was used according to our published protocols [31]. On the first day of the Morris water maze, the rats were trained to swim in a pool filled with opaque water (23–25 °C) to a visible platform approximately 1 cm above the surface of the water and remain on the platform for 20 s until removed by the operator. The following three days (day 2–4 of testing) consisted of 3 trials/day with a 10 min inter-trial interval per rat to train the rats to locate a submerged target platform (learning phase). For each trial, a rat was placed into the pool at a randomly assigned point, equidistant from the target. Rats were allowed 90 s to locate the target with a trial ending when either the rat located the target and climbed onto it, or 90 s had passed. Once the target was located, each rat was allowed to sit on the platform and observe visual cues placed on the walls to aid in the formation of spatial memory for 20 s. After the 20 s passed, the rat was removed from the water maze and placed into a carrier to dry and wait for the next trial. Rats that did not locate the hidden target were guided to the target by means of the operator tapping on the target until the rat swam to and climbed onto the platform. Twenty seconds were then provided for the rat to observe spatial cues and rest, and then the rat was returned to the carrier. The target remained in the same location throughout all 3 days of training. On day 5, each rat was administered a probe trial to test for spatial memory retention. During the probe trial, the underwater platform was removed. Each rat was placed into the water at one of the pre-determined random entry points and allowed 30 s to swim to the target location and search for the platform. At the end of 30 s, the platform was returned to its original location and the rat was allowed 20 s to sit on the platform to reduce stress. Latency and pathlength to the target were recorded using ANY-maze software (v. 5.14, Stoelting Co.). Latency and pathlength to the target during the probe trial were used as indicators of spatial memory retention, latency on each day leading up to the probe trial was used to measure learning. A learning index was generated by averaging the latency to the target of the all trials (3/day) of days 2 through 4 to examine performance during the learning phase [64].

### Sample collection

To collect tissue and blood samples, rats were anesthetized with isoflurane (2–3%) and decapitated during the first 2 h of the rats’ active phase of the circadian rhythm [9, 31, 65]. Trunk blood was collected in EDTA tubes, and then centrifuged at 2000×*g* for 10 min at 4 °C to collect plasma. Plasma was stored at − 80 °C. Each brain was quickly removed, flash frozen in 2-methlylbutane, and sliced into 1-mm coronal sections using a brain matrix (RBM-4000C, ASI Instruments, Warren, MI). Using blunt 20-guage needles attached to 1 ml syringes for brain region microdissection [9, 31, 65–67], the dentate gyrus (DG) and CA1 region of the dorsal hippocampus (− 5.30 mm from Bregma) were isolated according to Paxinos and Watson’s brain atlas [68]. Micro-dissected brain samples were placed into microcentrifuge tubes to be stored at − 80 °C until protein analysis.

### Hormone assays

Steroid hormones were extracted from plasma via acetonitrile preparation [69]. Briefly, plasma samples were diluted 2:3 in HPLC grade acetonitrile, then vortexed at room temperature. After 10 min, the plasma samples were centrifuged at 17,000×*g* at 4 °C for 5 min. Supernatants were collected and dried by vacuum centrifugation. All samples were reconstituted in the appropriate assay buffer prior to use in ELISA assay according to manufacturer’s instructions. Commercially available, competitive immunoassay ELISA kits were used for quantitative determination of circulating testosterone (ADI-900-065, Enzo Life Sciences Inc., Farmingdale, NY), corticosterone (ADI-900-097, Enzo Life Sciences Inc., Farmingdale, NY), and estradiol (RTC009R, BioVendor, Czech Republic) according to manufacturer's instructions [70, 71]. The intra-assay coefficient of variation for testosterone ELISA was 7.8% with an inter-assay coefficient of variation at 9.3%. The sensitivity of this testosterone ELISA was 5.67 pg/ml at the 2-standard deviation (s.d.) confidence limit. The intra-assay coefficient of variation for corticosterone ELISA was 6.6% with an inter-assay coefficient of variation at 7.8%. The sensitivity of the corticosterone ELISA was 26.99 pg/ml at the 2 s.d. confidence limit. The intra-assay coefficient of variation for estradiol ELISA was 6.1% with an inter-assay coefficient of variation at 7.0%. The sensitivity of estradiol ELISA was 2.5 pg/ml at the 2 s.d. confidence limit. Reported values are mean ± s.d.

### Tissue sample preparation, electrophoresis, and western blots

For protein analysis, frozen tissue samples were thawed in RIPA buffer (VWR, cat #N653) containing (per 0.5 ml): 2.5 µl Halt™ protease and phosphatase inhibitor (Thermo Scientific, cat #78,442), 1 µl 0.5 M ethylenediaminetetraacetic acid (EDTA, Sigma-Aldrich), and 1 µl 0.5 mM dithiothreitol (DTT, Sigma-Aldrich) for homogenization, as previously described [9, 67, 72–74]. Total protein concentration levels in the homogenate were determined using Pierce BCA Protein Assay (Thermo Fisher, cat #23225). Samples were denatured with β-mercaptoethanol and boiled at 100 °C for 5 min. Equal volumes of denatured tissue samples containing 30 µg protein were loaded into a Bio-Rad 4–15% polyacrylamide gel. The gel then underwent electrophoresis at 25 milliamps (mA) in a tris–glycine running buffer followed by overnight transfer at 4 °C onto a PVDF membrane at 50 mA. Following 30 min washing, membranes were blocked for 30 min with 5% nonfat milk in tris-based saline (TBS)-Tween (TBST) at room temperature. Membranes were then transferred to 1% nonfat milk TBST solutions containing specific primary antibodies and incubated overnight at 4 °C. Primary antibodies used on CA1 hippocampal micro-punched tissue included DAT-HRP (Santa Cruz, SC-32259 1:5000), MAO-A (Abcam, ab126751 1:1000), NR2A (NeuroMab, N327A/38 1:500). For DG tissue samples, primary antibodies for doublecortin (Santa Cruz, sc-271390 1:500) and EGR-1 (Santa Cruz, sc-515830 1:500) were used. Afterwards, membranes were washed in 10-min increments for 30 min, and then incubated in 1% milk TBST- secondary antibody solutions including HRP-conjugated horse anti-mouse (Cell Signaling, 7076P2 1:2500) or HRP-conjugated goat anti-rabbit (Cell Signaling, 7074P2 1:5000) at room temperature for 1 h. For protein normalization, we used beta-actin primary antibody (GeneTex, GTX 629630 1:3000), which was incubated for 1 h at room temperature. Protein bands were visualized using West Pico (Thermoscientific, cat #34580) or West Fempto (Thermoscientific, cat #34095) enhanced chemiluminescence detection using a Syngene G-Box Chemi XRQ system with GeneSys software (version 1.5.2.0) as previously described [74]. NIH Image J software (version 1.48v) was used to quantify band densitometry of protein of interest normalized to beta actin band densitometry. The following proteins of interest were quantified: (1) glutamatergic NMDA receptors (NR2A) that mediate excitatory neuronal synaptic activity and are associated with spatial memory and social behaviors [75, 76]; (2) dopamine transporter (DAT), a monoamine neurotransmitter transporter [77] that can impact memory function, anxiety, and social function [78–80]; (3) monoamine oxidase-A (MAO-A), an enzyme that catalyzes monoamines (e.g., serotonin, norepinephrine, and dopamine) and is associated with memory and social function [80–82]; (4) early growth response protein 1 (EGR-1), a transcription factor expressed in numerous cell types that acts to mediate cellular activity [83, 84], with hippocampal expression associated with memory, anxiety and neuropsychiatric disorders [85, 86]; and (5) doublecortin (DC), a neurogenesis marker associated with memory, anxiety and social function [87–90].

### Statistical analysis

Significance was defined as p ≤ 0.05. Statistical analyses were conducted in IBM SPSS (SPSS v. 29.0.0, IBM, 2022). Data distribution was tested using the Shapiro–Wilk test. Data with non-Gaussian distribution were normalized by square root transformation through the SPSS transformation function (x = sqrt(x)). Outliers greater than 2 standard deviations from the mean were removed from analysis. Since social withdrawal was exhibited by a low number of animals, Fisher’s exact test was used to determine the impact of gestational CIH on social withdrawal [91]. For all other behavior tests, ELISAs, and protein analyses, the main effects and interactions were examined using two-way ANOVAs with the factors of treatment and sex within either the puberty or young adult groups, wherein we provide the F values, degrees of freedom, p-values, and η^2^ (measure of effect size). To examine the effect of age, two-way ANOVAs with the factors of treatment and age within either male or female groups were performed. Following ANOVA, a Fisher’s LSD posthoc test was used to determine specific group differences. Results are presented as mean ± S.E.M. unless otherwise indicated.

## Results

### Late gestational CIH induced social impairments in female offspring without affecting aggressive or exploratory behaviors

**Fig. 1:**
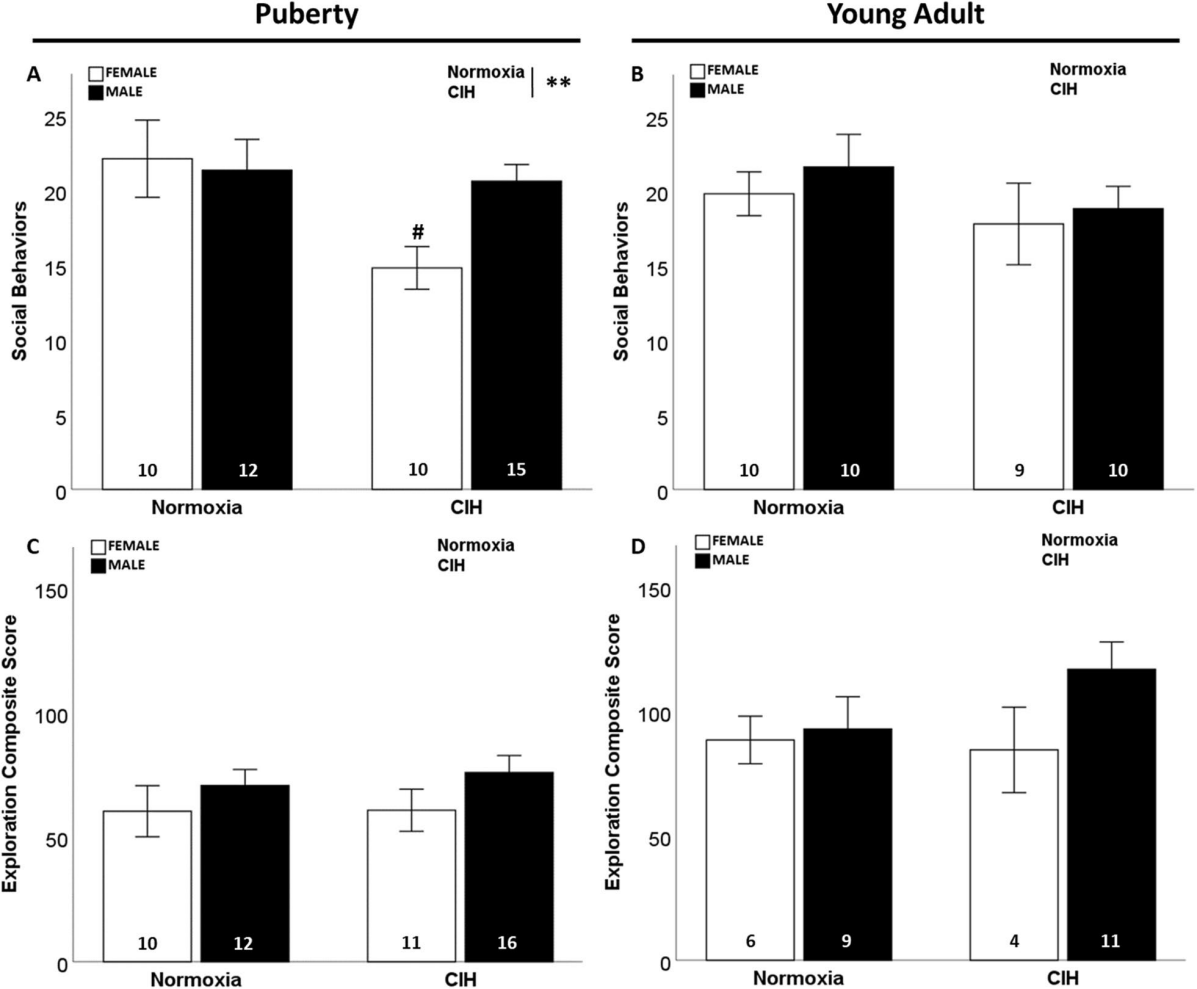
Social and exploratory behaviors. Gestational CIH decreased social behaviors in female rats during puberty (**A**) but not during young adulthood (**B**). No effect of gestational CIH was observed on male rats during puberty (**A**) or young adulthood (**B**). No effect of sex was observed on social behaviors (A, B). No effect of gestational CIH or sex was observed in exploratory behavior (**C**, **D**). Analyzed by Two-way ANOVA with Fisher’s LSD multiple comparisons tests. ANOVA significance indicated by: ** = CIH; Post-hoc significance indicated by: # versus normoxic female; p ≤ 0.05

We observed that gestational CIH significantly reduced social behaviors displayed by pubertal rats (F_1, 43_ = 4.934; p = 0.032; η^2^ = 0.093; Fig. 1A), especially in pubertal females (p ≤ 0.05). However, this effect of gestational CIH on social behaviors was not maintained in young adult rats (Fig. 1B). No effects of sex or age were observed on social behaviors.

We observed no effects of gestational CIH or sex on exploratory behavior in pubertal or young adult rats (Fig. 1C, D). We found increased exploratory behavior by young adult females compared to pubertal females (F_1, 27_ = 5.001; p = 0.034; η^2^ = 0.156; Additional file 2: Fig. S2A). This effect was also observed in young adult males compared to pubertal males (F_1, 44_ = 11.943; p = 0.001; η^2^ = 0.200; Additional file 2: Fig. S2B).

**Fig. 2:**
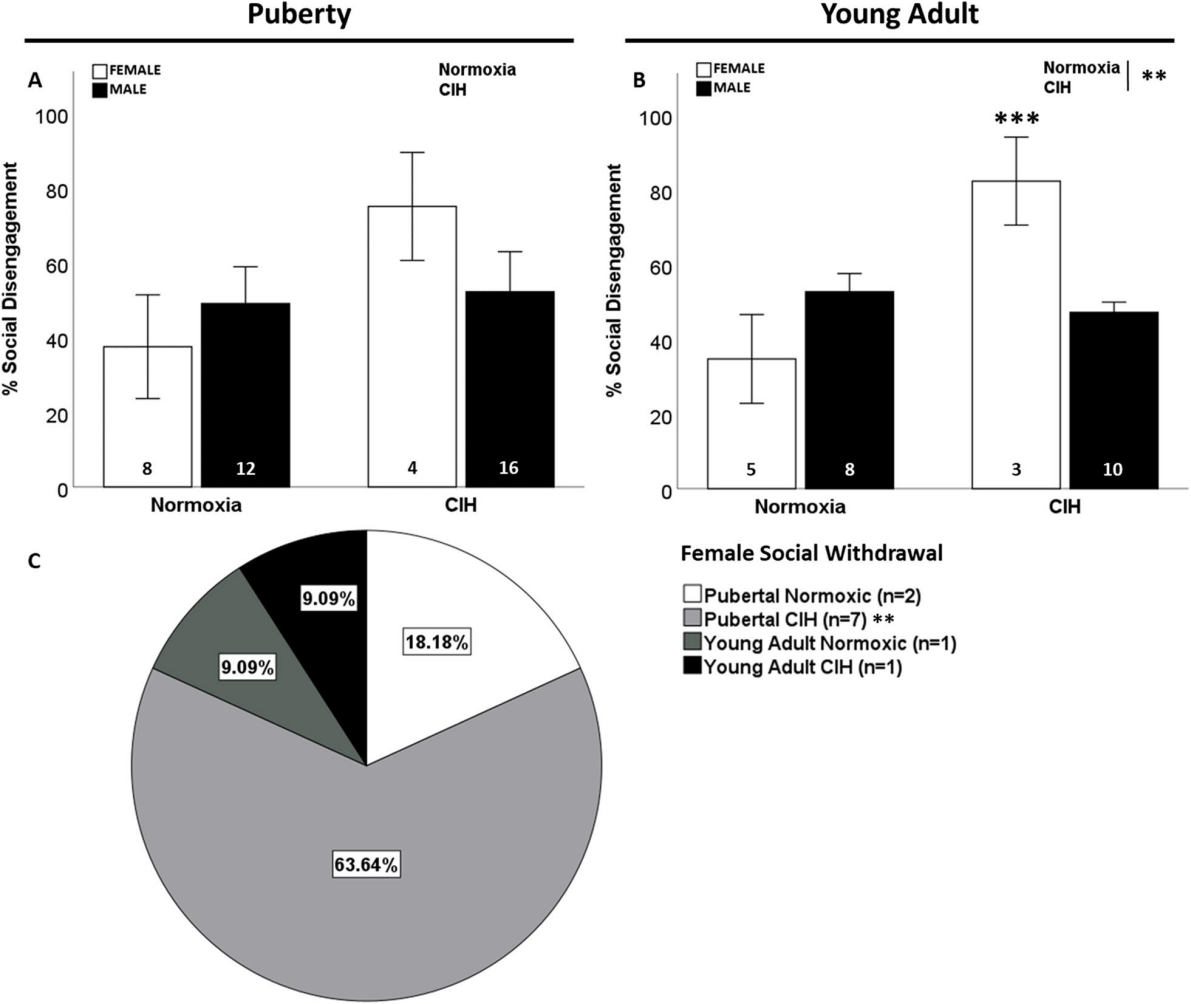
Social disengagement. No effect of gestational CIH or sex was observed on social disengagement in pubertal rats (**A**). Gestational CIH only increased percentage of young adult female rats that exhibited social disengagement (**B**). Social withdrawal was significantly associated with gestational CIH only in pubertal females (**C**). Social disengagement analyzed by Two-way ANOVA with Fisher’s LSD multiple comparisons tests (**A**, **B**). Relationship between social engagement and CIH measured by Fisher’s exact test (**C**). Significance indicated by: ** = CIH, *** = interaction; p ≤ 0.05

No effects of gestational CIH or sex on social disengagement were observed in pubertal rats (Fig. 2A). In contrast, there was a significant interaction between gestational CIH and sex on social disengagement in young adult rats (F_1, 26_ = 14.472; p < 0.001; η^2^ = 0.307; Fig. 2B), in which gestational CIH increased social disengagement in young adult females. Further, late gestational CIH increased social disengagement in pubertal and young adult females (F_1, 20_ = 7.540; p = 0.014; η^2^ = 0.319; Additional file 3: Fig. S3A). No effect of CIH or age was observed in male offspring (Additional file 3: Fig. S3B). Late gestational CIH increased social withdrawal in pubertal females (p ≤ 0.05; Fig. 2C). No males displayed social withdrawal.

Since developmental dysfunction and altered steroid hormones can impact aggression in pubertal and young adult rats [54, 60–62], aggressive behaviors were recorded. Aggressive behaviors include kicking, biting, dominance posture, boxing and fighting [60–62]. Offspring displayed little to no aggressive behaviors, regardless of gestational CIH, sex, or age (Additional file 10: Table S2).

### Late gestational CIH increased repetitive behaviors only in pubertal females

**Fig. 3:**
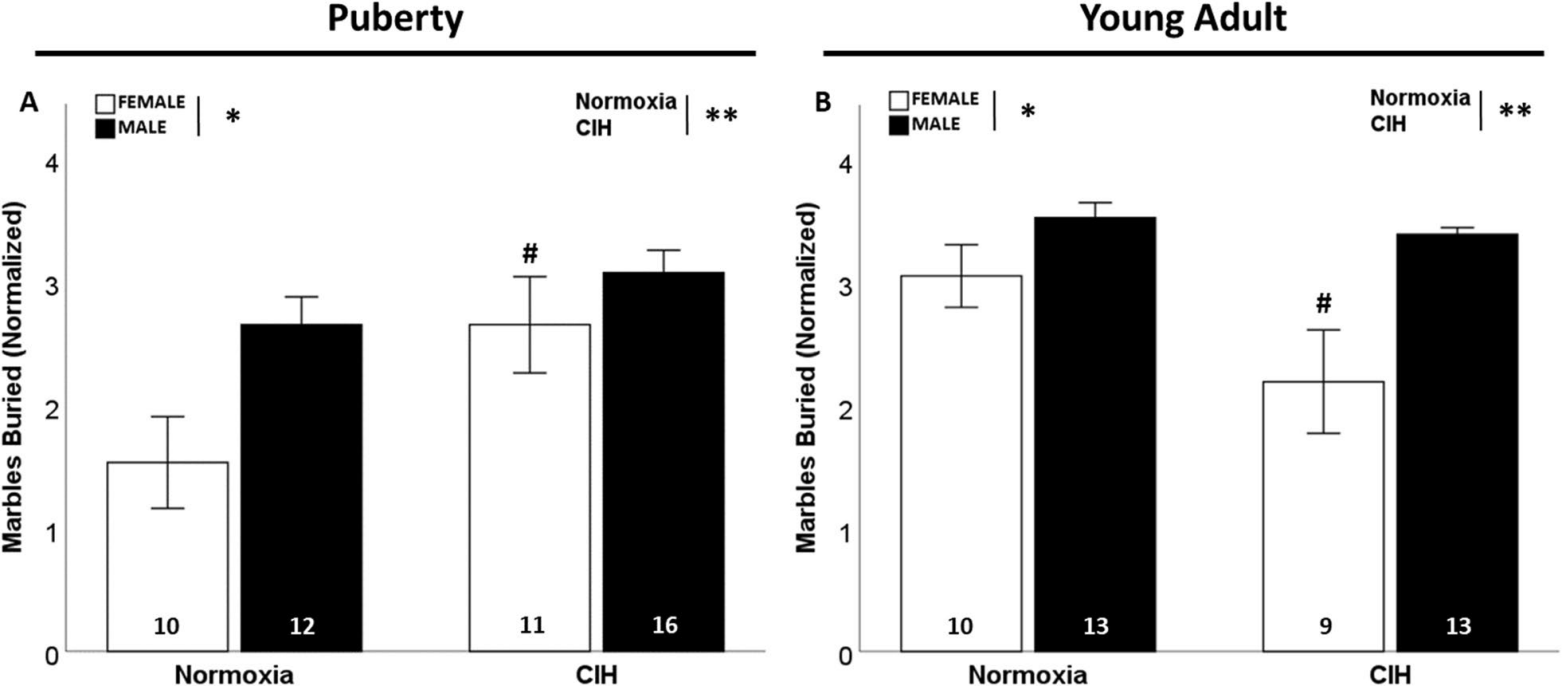
Repetitive behaviors. Gestational CIH increased marble burying in pubertal females (**A**) and young adult females (**B**). Males buried more marbles than females, regardless of age (**A**, **B**). Normalized by square-root transformation (**A**, **B**). Analyzed by Two-way ANOVA with Fisher’s LSD multiple comparisons tests. ANOVA significance indicated by: * = sex, ** = CIH; Post-hoc significance indicated by: # versus normoxic female, p ≤ 0.05

In pubertal rats, there was a significant effect of gestational CIH on marble burying (F_1, 49_ = 7.215; p = 0.010; η^2^ = 0.118; Fig. 3A), where gestational CIH exposed pubertal females buried more marbles than normoxic pubertal females (p ≤ 0.05). There was also a significant effect of sex on marble burying (F_1, 49_ = 7.205; p = 0.010; η^2^ = 0.118; Fig. 3A) in pubertal rats, where males buried more marbles than females. Conversely, in young adulthood the significant effect of CIH (F_1. 45_ = 5.204; p = 0.028; η^2^ = 0.082; Fig. 3B) is reversed, with fewer marbles buried by offspring exposed to gestational CIH, specifically by females (p ≤ 0.05). Similar to puberty, males during young adulthood (F_1, 45_ = 14.761; p < 0.001; η^2^ = 0.232; Fig. 3B) buried more marbles than females. There was a significant interaction between gestational CIH and age in females (F_1, 36_ = 7.322; p = 0.010; η^2^ = 0.161; Additional file 4: Fig. S4A), in which gestational CIH increased repetitive behaviors only in pubertal females. Age also affected marble burying in males, as young adult males buried more marbles than pubertal males (F_1, 50_ = 13.669; p < 0.001; η^2^ = 0.202; Additional file 4: Fig. S4B).

### Late gestational CIH did not induce anxiety-like behaviors

**Fig. 4:**
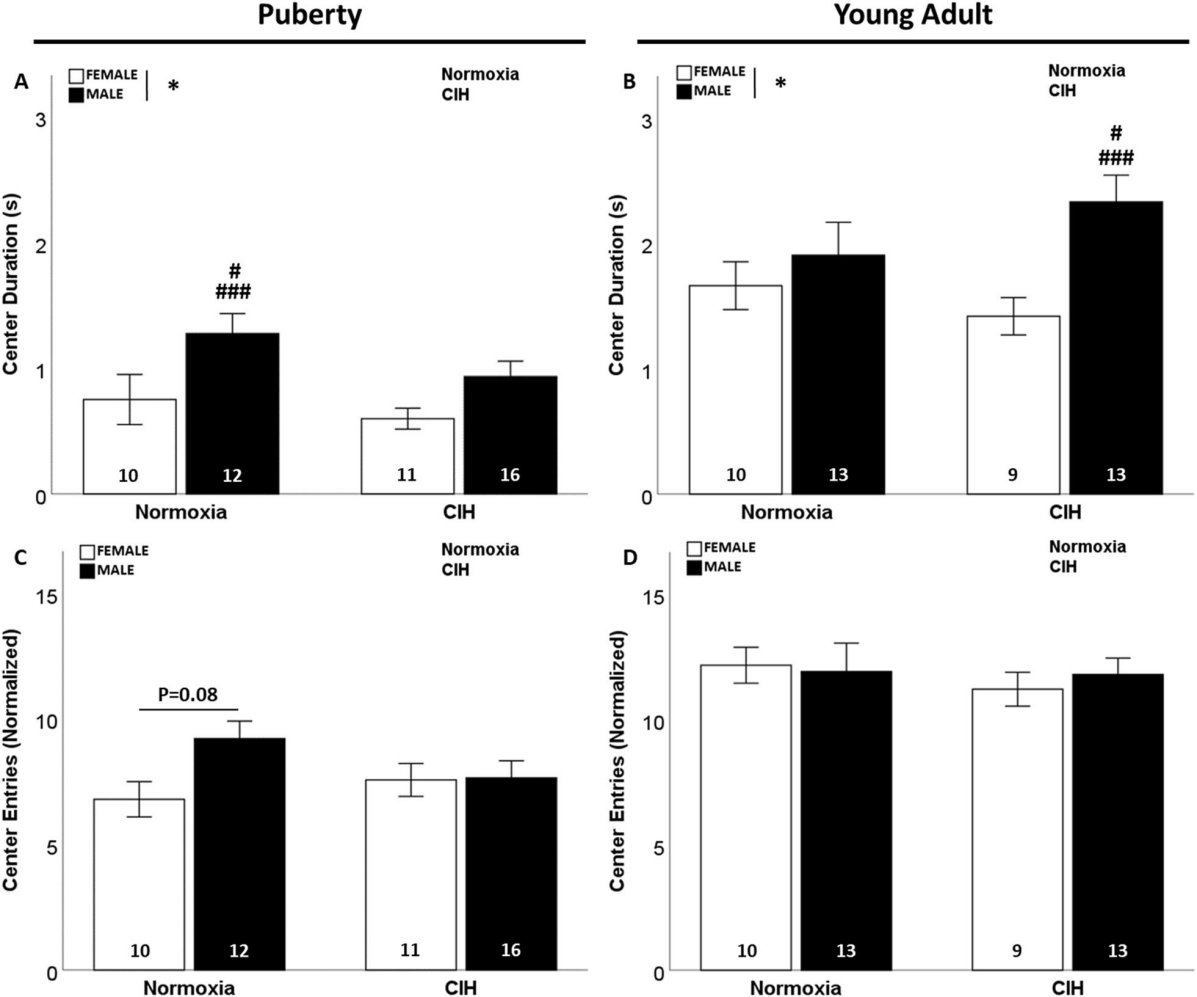
Anxiety-like behaviors. Females spent less time in the center of the open field compared to males, irrespective of age or gestational CIH (**A**, **B**). No effect of gestational CIH or sex was observed on center entries, regardless of age (**C**, **D**). Normalized by square-root transformation (**C**, **D**). Analyzed by Two-way ANOVA with Fisher’s LSD multiple comparisons tests. ANOVA significance indicated by: * = sex; Post-hoc significance indicated by: # versus normoxic female, ### versus CIH female; p ≤ 0.05

No effect of gestational CIH was observed on center duration in pubertal offspring (Fig. 4A) or young adult offspring (Fig. 4B). Pubertal males spent more time in the center of the open field arena than females during puberty (F_1, 45_ = 8.759; p = 0.005; η^2^ = 0.153; Fig. 4A), in which normoxic males spent more time than normoxic or gestational CIH exposed females (p ≤ 0.05). Young adult males spent more time in the center than females (F_1, 41_ = 5.736; p = 0.021; η^2^ = 0.113; Fig. 4B), wherein gestational CIH exposed males spent more time in the center than normoxic or gestational CIH exposed females (p ≤ 0.05). There was a significant effect of age on time spent in the center of the open field, where young adult females (F_1, 36_ = 29.056; p < 0.001; η^2^ = 0.436; Additional file 5: Fig. S5A) and males (F_1, 50_ = 25.264; p < 0.001; η^2^ = 0.311; Additional file 5: Fig. S5B) spent more time than pubertal rats. Gestational CIH exposed young adult males spent more time in the center than either normoxic males or gestational CIH exposed pubertal males (p ≤ 0.05). We observed no effects of gestational CIH or sex on the number of center entries displayed by offspring (Fig. 4C, D). Similar to center duration, we observed that aging is associated with increased center entries, as shown by more entries performed by young adult females (F_1, 36_ = 42.528; p < 0.001; η^2^ = 0.531; Additional file 5: Fig. S5C) and young adult males (F_1, 50_ = 17.729; p < 0.001; η^2^ = 0.255; Additional file 5: Fig. S5D) compared to pubertal rats.

### Late gestational CIH only impacted spatial memory function in pubertal male rats

**Fig. 5:**
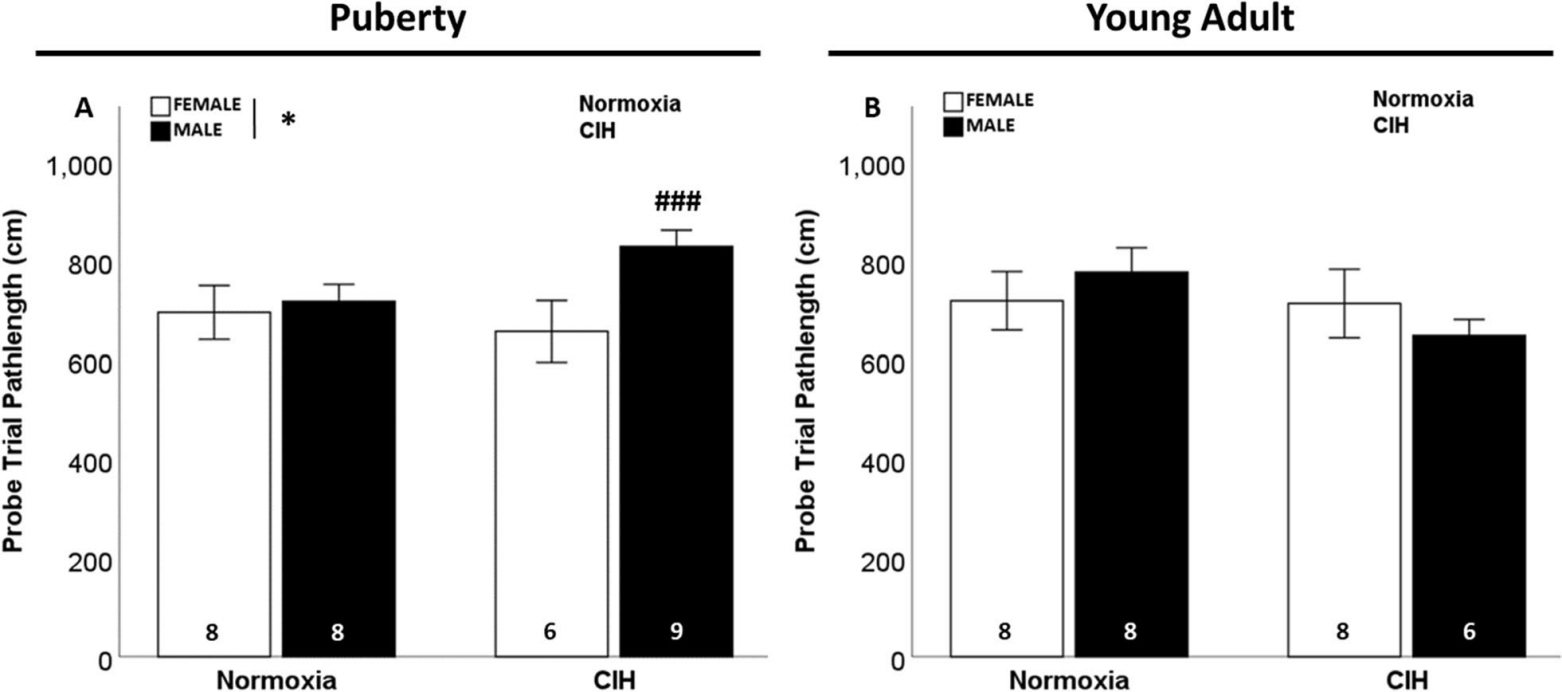
Spatial memory. Pubertal males had longer pathlength to target during Morris water maze probe trial (**A**), with the sex difference observed primarily in gestational CIH exposed pubertal males. No effect of sex or gestational CIH was observed on pathlength in young adult rats (**B**). Analyzed by Two-way ANOVA with Fisher’s LSD multiple comparisons tests. ANOVA significance indicated by: * = sex; Post-hoc significance indicated by: ### = versus CIH female; p ≤ 0.05

We observed sex differences in pathlength to the target, in which pubertal females exhibited shorter pathlength to the target than pubertal males (F_1, 27_ = 4.495; p = 0.043; η^2^ = 0.129; Fig. 5A). However, gestational CIH did not affect pathlength to the target in pubertal (Fig. 5A) or young adult offspring (Fig. 5B). No sex differences in spatial memory were observed in young adult rats. No effect of age on spatial memory was observed in females (Additional file 6: Fig. S6A). In male offspring, the effects of gestational CIH on spatial memory were dependent on age. Specifically, gestational CIH increased pathlength to the target in pubertal males, but decreased pathlength in young adult males (F_1, 27_ = 9.375; p = 0.005; η^2^ = 0.242; Additional file 6: Fig. S6B). Although we observed significant effects of gestational CIH, sex, and age on spatial memory-associated pathlength to target, we did not observe any effects on latency to the target during the probe trial (Additional file 11: Table S3).

**Fig. 6:**
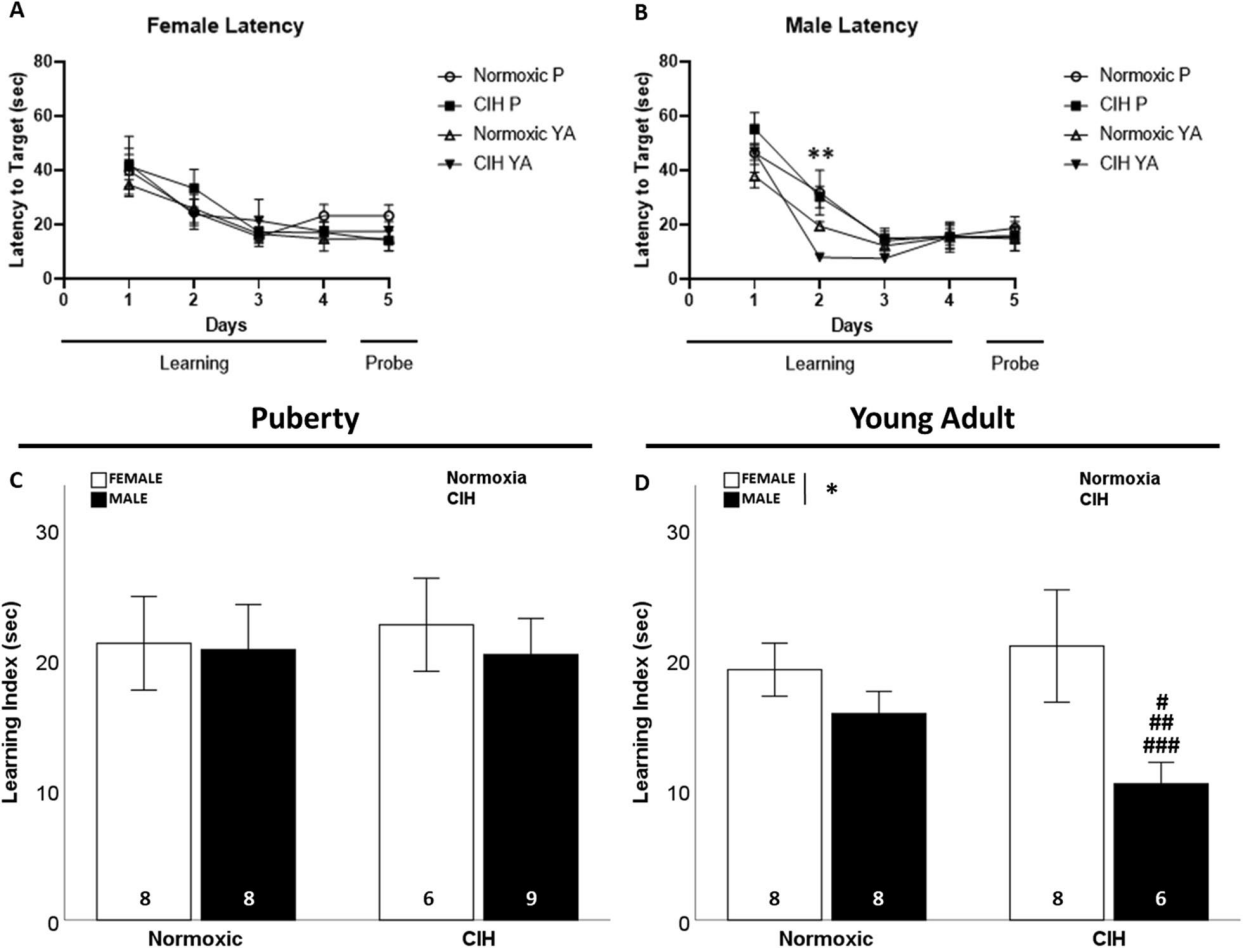
Spatial learning. No effect of gestational CIH on latency to target was observed in females, regardless of age (**A**). Age improved latency to target during day 2 of learning in males with no effect of age observed on other days (**B**). Learning index was not affected by gestational CIH or sex during puberty (**C**). Males had shorter learning index than females during young adulthood (**D**). Analyzed by Two-way ANOVA with Fisher’s LSD multiple comparisons tests. ANOVA significance indicated by: * = sex; ** = age; Post-hoc significance indicated by: # = versus normoxic female, ## = versus normoxic male; ### = versus CIH female; p ≤ 0.05; *P* = *Puberty*;* YA* = *Young Adult*

There were no sex differences or effects of gestational CIH on learning (latency to target) in offspring (Fig. 6A, B). However, we did observe faster learning (shorter latency to target) in young adult males on day 2 of training compared to pubertal males (F_1, 27_ = 12.109; p = 0.002; η^2^ = 0.290; Fig. 6B) or young adult females (F_1, 26_ = 6.824; p = 0.015; η^2^ = 0.187), though this was not observed on any other days of testing. This effect of age on learning (i.e., shorter latency to target) was not observed in female offspring (Fig. 6A). There were no effects of sex or CIH on the learning index in pubertal rats (Fig. 6C). However, we did observe that young adult males had a shorter learning index than young adult females (F_1, 26_ = 6.154; p = 0.020; η^2^ = 0.180; Fig. 6D). No effects of age were observed on the learning index, regardless of sex.

### Late gestational CIH did not impact expressions of proteins associated with social and cognitive function within the dorsal hippocampus

Of the proteins that we examined, gestational CIH did not impact protein expression (Table 1; Additional file 7: Fig. S7; Additional file 8: Fig. S8), indicating that gestational CIH exposure from GD 15–19 does not impact cellular function in the dorsal hippocampus of offspring. Although we did not observe any effects of late gestational CIH on dorsal hippocampal cellular activity, there were sex– and age-dependent effects on DAT, NMDA receptors, and EGR-1 protein expression (Table 1). Sex only affected DAT expression in the CA1, where young adult males had significantly higher expression compared to young adult females (F_1, 20_ = 8.122, p = 0.010, η^2^ = 0.277). We also observed age differences: (1) decreased DAT (F_1, 20_ = 4.484, p = 0.047, η^2^ = 0.179) and decreased NR2A (F_1, 20_ = 6.716, p = 0.017, η^2^ = 0.251) in the CA1 of young adult females compared to pubertal females, (2) decreased EGR-1 (F_1, 20_ = 17.013, p < 0.001, η^2^ = 0.450) in the dentate gyrus of young adult females compared to pubertal females, and (3) decreased EGR-1 (F_1, 20_ = 15.800, p < 0.001, η^2^ = 0.424) in the dentate gyrus of young adult males compared to pubertal males.

**Table 1:** Expression of proteins associated with social and cognitive function within the dorsal hippocampus

		Normoxia			CIH		
			**Puberty**	Young adult**		**Puberty**	Young adult **
CA1	Dopamine Transporter (DAT)	F	91.27 ± 29.25	71.10 ± 18.23 ^##^	F	104.42 ± 45.78	68.21 ± 31.17 ^##^
		M *	84.76 ± 26.75	90.48 ± 25.92 ^#^	M*	92.81 ± 13.74	105.94 ± 20.84 ^#^
			**Puberty**	**Young adult**		**Puberty**	**Young adult**
	Monoamine Oxidase A (MAO-A)	F	49.33 ± 13.76	52.03 ± 45.22	F	53.28 ± 14.38	43.49 ± 34.70
		M	58.68 ± 38.36	54.45 ± 42.02	M	55.20 ± 25.98	43.79 ± 51.97
			**Puberty**	Young adult**		**Puberty**	Young adult**
	NMDA Receptor 2A (NR2A)	F	75.11 ± 14.95	54.20 ± 19.37^##^	F	76.22 ± 27.92	51.17 ± 22.55^##^
		M	73.82 ± 13.56	70.74 ± 24.76	M	64.43 ± 18.25	60.33 ± 25.83

		Normoxia			CIH		
DG			**Puberty**	**Young adult**		**Puberty**	**Young adult**
	Doublecortin (DC)	F	99.27 ± 43.29	70.64 ± 45.29	F	79.45 ± 20.34	75.99 ± 46.04
		M	94.30 ± 40.15	77.03 ± 31.08	M	103.62 ± 31.93	85.80 ± 23.07
			**Puberty**	Young adult**		**Puberty**	Young adult**
	Early Growth Response 1 (EGR-1)	F	106.71 ± 34.31	57.76 ± 28.22^##^	F	94.73 ± 19.25	49.51 ± 28.00^##^
		M	107.57 ± 26.71	59.96 ± 27.87^##^	M	117.85 ± 10.63	76.40 ± 37.54^##^

All values represent percentage of Beta Actin expression. All values presented as mean ± SD. Analyzed by Two-way ANOVA with Fisher’s LSD multiple comparisons tests, n = 6/group. ANOVA significance within treatment group (i.e., Normoxia or CIH) indicated by: * = sex, ** = age; Post-hoc significance within treatment group (i.e., Normoxia or CIH) indicated by: ^#^ = versus female, ^##^ = versus puberty; p ≤ 0.05

### Late gestational CIH differentially affected circulating corticosterone levels in females

We observed sex differences in circulating testosterone and corticosterone levels, as well as an effect of gestational CIH on corticosterone levels (Table 2). No effect of gestational CIH, sex, or age was observed on plasma estradiol levels (Table 2). No effect of gestational CIH was observed on plasma testosterone levels. However, we observed expected sex differences in testosterone levels: (1) higher testosterone levels in pubertal males compared to pubertal females (F_1, 32_ = 30.166; p < 0.001; η^2^ = 0.480) and (2) higher testosterone levels in young adult males compared to young adult females (F_1, 35_ = 41.037; p < 0.001; η^2^ = 0.526). Testosterone levels also increased with age in females (F_1, 31_ = 10.739; p = 0.003; η^2^ = 0.235) and males (F_1, 36_ = 31.029; p < 0.001; η^2^ = 0.462). The testosterone [92, 93] and estradiol levels [94–97] observed in this study are consistent with other studies.

**Table 2:** Plasma concentration of steroid hormones

	Normoxia			CIH		
		**Puberty**	**Young Adult**		**Puberty**	**Young Adult**
Corticosterone (ng/ml)	F	307.46 ± 107.24	322.22 ± 63.38	F	457.58 ± 203.25***	246.75 ± 63.89^###^
	M **	168.94 ± 74.95 ^##^	168.32 ± 39.67^##^	M **	218.80 ± 92.28^##^	160.03 ± 57.16^##^

	Normoxia			CIH		
		**Puberty**	**Young Adult**		**Puberty**	**Young Adult**
Estradiol (pg/ml)	F	26.70 ± 4.11	55.15 ± 48.92	F	40.85 ± 26.79	67.80 ± 42.65
	M	42.74 ± 58.77	43.12 ± 30.50	M	39.99 ± 35.31	38.44 ± 18.53

	Normoxia			CIH		
		**Puberty**	Young Adult**		**Puberty**	Young Adult**
Testosterone (ng/ml)	F	0.51 ± 0.30	0.99 ± 0.48	F	0.70 ± 0.31	1.52 ± 0.95 ^###^
	M **	1.52 ± 0.67 ^##^	2.92 ± 1.08^##, ###^	M**	1.62 ± 0.62^##^	2.99 ± 0.69^##, ###^

All values presented as mean ± SD without normalization. All values normalized by square root transformation for analysis. Analyzed by Two-way ANOVA with Fisher’s LSD multiple comparisons tests. Corticosterone: n = 8–10/group; Estradiol: n = 5–9/group; Testosterone: n = 7–10/group. ANOVA significance within treatment group (i.e., Normoxia or CIH) indicated by: * = CIH, ** = sex or age, *** = interaction of age/CIH; Post-hoc significance within treatment group (i.e., Normoxia or CIH) indicated by: ^#^ = versus normoxia, ^##^ = versus female, ^###^ = versus puberty; p ≤ 0.05

To examine the impact of gestational CIH on circulating hormones associated with the stress response, we quantified plasma corticosterone levels (Table 2). Gestational CIH increased circulating corticosterone in pubertal rats (F_1, 31_ = 5.407; p = 0.027; η^2^ = 0.094), particularly in female offspring (p ≤ 0.05). A sex difference was also observed in pubertal rats, with higher plasma concentrations of corticosterone observed in female offspring than male offspring (F_1, 31_ = 19.247; p < 0.001; η^2^ = 0.338). In young adulthood, the effect of gestational CIH on circulating corticosterone levels was reversed, with lower corticosterone observed (F_1, 35_ = 5.313; p = 0.027; η^2^ = 0.061), specifically in young adult females (p ≤ 0.05). The sex difference in corticosterone levels was maintained into young adulthood, with higher levels of corticosterone observed in young adult female offspring than young adult male offspring (F_1, 35_ = 43.840; p < 0.001; η^2^ = 0.501). In female offspring, an interaction between age and gestational CIH was observed, with plasma corticosterone levels increasing in gestational CIH exposed pubertal offspring but decreasing in gestational CIH exposed young adult offspring (F_1, 33_ = 8.343; p = 0.007; η^2^ = 0.172). No effect of age on corticosterone levels was observed in male offspring.

## Discussion

The major findings of this study are the effects of late gestational hypoxia on offspring are dependent on sex and age. In pubertal female offspring, we observed social function impairments, increased repetitive behaviors, and corticosterone dysregulation. These CIH-induced social impairments and corticosterone dysregulation in pubertal females were sustained into young adulthood. In contrast, late gestational CIH induced a different behavioral phenotype in pubertal males in which cognitive dysfunction (increased pathlength) was observed in the absence of anxiety-like behaviors, aggression, and altered exploratory behaviors. The only long-term effect of gestational CIH exposure in male offspring was impaired 50 kHz USVs in young adult males that were identified in our previous study [9].

This study is the first to examine the effects of late gestational CIH on social function, anxiety, and cognitive functions in rat offspring. Further, this is the only study to use Long-Evans rats, as prior studies used either Sprague Dawley or Wistar rats (Additional file 12: Table S4). We chose to use Long-Evans rats in our studies, as this rat strain exhibits increased behaviors (activity, cognitive function, exploration), greater stress reactivity, and increased sensitivity to hypoxia compared to other rat strains [71, 98, 99]. Additionally, Long-Evans rats are commonly used to study prenatal brain development [100–102]. Brain development during pregnancy occurs in three stages (Additional file 12: Table S4): (1) stage 1 (GD 1–10) in which the neural tube is formed and is comparable to the first three weeks of human gestation [103, 104]; (2) stage 2 (GD 10–15) in which the establishment of cortical and subcortical brain regions occurs and is comparable to the first 2 months of human gestation [41, 105, 106]; and (3) stage 3 (GD 15–22) in which cortical and subcortical brain maturation occurs and is comparable to the last 7–8 months of human gestation [41, 104, 107]. We have previously shown that short-term, late gestational CIH (GD 15–19) had sex- and age-specific effects on nigrostriatal pathway maturation in pubertal female and young adult male offspring [9]. Impairment of the nigrostriatal pathway is associated with multiple neuropsychiatric disorders, such as cognitive dysfunction [108], autism spectrum disorder [108, 109], and mood disorders [108, 109]. Therefore, here we examined the impact of late gestational CIH on social function, anxiety, and cognitive function in offspring.

Prior studies investigating the impact of gestational hypoxia in rodent offspring (Additional file 12: Table S4) have found equivocal findings that range from social impairments in male offspring with no effects in female offspring [20, 24] to social impairments in female offspring with no effects in male offspring [22, 23] or social impairments in both sexes [21]. Reports of sex differences in cognition due to gestational hypoxia have been observed, with impairment observed in male offspring [20, 110], female offspring [111], or neither sex [21, 22, 112]. These behavioral differences in offspring exposed to gestational hypoxia may be due to the timing or the type of hypoxic insult, as they ranged in hypoxia exposure duration (1–21 days), hypoxia cycles (intermittent, sustained hypoxia), and intensity of lowered oxygen concentration (13%-5%). Our study utilized CIH (10 hypoxia cycles/hour/for 8 h each day at 10% O_2_) during late gestation (GD 15–19). Although no other study has used gestational intermittent hypoxia exposure targeting brain development stage 3, prior studies using a sustained hypoxia protocol during this time have been conducted (Additional file 12: Table S4). Specifically, sustained hypoxia during this time period (GD 15–22) impaired social behaviors displayed by young adult female offspring and not young adult male offspring [22, 23], had no impact on anxiety-like behaviors in young adult male and female offspring [22, 113], and decreased repetitive behaviors in young adult female offspring [23]. However, there were differences between these prior studies and our current study, in which sustained hypoxia impaired cognitive function in young adult female offspring [111], decreased repetitive behaviors in young adult male offspring [22, 23], and increased anxiety-like behaviors in young adult male offspring [22, 23], indicating that the type of hypoxic exposure during this critical period of brain development is important.

This study is the first to examine the impact of sex as a biological variable in behavior displayed by pubertal offspring exposed to short-term hypoxic stress during late gestation. We observed several sex- and age-associated developmental effects on brain and behavior induced by short-term CIH during late gestation. A recent study by Vanderplow et al. [20] showed that long-term CIH exposure from GD 10–21 induced functional effects in male offspring (impaired memory and social function in pubertal and adult males) but not female offspring. In contrast, our results using a short-term CIH protocol during late gestation (GD 15–19) found sex-specific effects in offspring that were dependent on age. Pubertal male offspring exposed to late gestational hypoxia exhibited cognitive impairment and suppressed 50 kHz USV calls [9], and this suppressed USV call production was sustained into young adulthood [9]. We observed a different phenotype in females. Pubertal female offspring exposed to late gestational hypoxia exhibited increased social dysfunction, increased repetitive behaviors, corticosterone dysregulation, and impaired 50 kHz USVs [9], with only social dysfunction and corticosterone dysregulation present during young adulthood. Although social dysfunction, repetitive behaviors, and corticosterone dysregulation have been associated with anxiety-like behavior in rats [114–118], we did not observe the presence of other anxiety-associated behaviors (e.g., open field center entries and duration, exploratory behavior, or stress-associated 22 kHz USVs [9, 45, 119]).

In our control animals that were not exposed to gestational CIH, we observed sex and age effects on repetitive behavior, anxiety-like behaviors, and corticosterone. Female rats displayed less repetitive behaviors (fewer marbles buried), increased anxiety-like behaviors (shorter open field center duration), and increased circulating corticosterone compared to male rats. Although the literature is equivocal on sex differences in anxiety-associated behaviors [22, 23, 113], multiple studies have shown that females exhibit higher corticosterone than males [120–123]. We found that aging decreased anxiety-like behaviors and increased exploratory behaviors in both males and females, consistent with other studies [124]. We also found sex-differences, in which we observed increased anxiety-like behaviors in females consistent with increased anxiety-like behavior previously reported in open field, light–dark box, and social interactions [58, 124]. However, studies have also shown that females exhibit decreased anxiety-like behavior in elevated plus mazes [58], indicating that testing conditions may impact this behavior. Indeed, it has been shown that several factors can influence anxiety-like behaviors, such as the testing apparatus (elevated plus maze, open field, social interactions, light–dark box) [58, 124]. Strain differences may mediate the equivocal findings on sex differences in center duration in an open field [58, 124, 125]. Prior studies that examined behaviors within the center of an open field range from no differences in center duration in Sprague Dawley rats [58] to increased center duration by Wistar female rats [125] and Long-Evans male rats [124]. Our study using Long-Evans rats is consistent with previous studies that showed increased center duration in Long-Evans rats that increased with age [124]. However, regardless of age, offspring in this study spent little time in the center of the open field, which could be an effect of arena size [125, 126].

Pubertal female offspring had shorter pathlengths in the Morris water maze than pubertal male offspring during the probe spatial memory trial, an effect that was not sustained into young adulthood. Prior studies have shown that females can exhibit shorter pathlength and latency, depending on experimental conditions (e.g., training duration and room lighting) [127]. Further, pubertal females have been observed to outperform pubertal males in hippocampal function tests, which may have contributed our observed sex differences [128, 129]. We observed no sex differences in the learning index by pubertal rats. In contrast, young adult males exhibited a shorter learning index compared to females, indicating better spatial learning performance. This sex difference in learning index is consistent with reports of improved learning performance in Morris water maze by males compared to females [130, 131].

In addition to behavioral effects of late gestational hypoxia, we examined glutamatergic (NR2A), dopaminergic (DAT), and serotonergic (MAO-A) associated proteins in the CA1 region of the dorsal hippocampus, as these have been associated with social and cognitive impairments [109, 132–135]. We also examined markers for cellular activity (EGR-1) and neurogenesis (doublecortin) within the dentate gyrus region of the dorsal hippocampus, as cellular activity and neurogenesis within the dentate gyrus is important in mediating spatial memory [136, 137] and social function [87–90]. Late gestational CIH did not impact protein expression within the CA1 (i.e., NR2A, DAT, MAO-A), the dentate gyrus (i.e., EGR-1, doublecortin), or the substantia nigra (i.e., calpain enzymatic activity, caspase-3 enzymatic activity, tyrosine hydroxylase), regardless of sex or age of the offspring [9]. While we observed expected fragments of NR2A that range from ~ 90 kDa to ~ 70 kDa (Neuromab), we showed no calpain cleavage of NR2A (~ 115 kDa [138, 139]), similar to no effect of gestational CIH on calpain cleavage of spectrin [9]. Future studies need to be conducted to examine the electrophysiological properties (long-term potentiation and long-term depression) of dorsal hippocampal neurons and neurogenesis (bromodeoxyuridine, BrdU), as dysfunction in these cellular properties have been linked with behavioral impairments [140, 141].

The dopamine system has been associated with anxiety-like behaviors [142, 143] and repetitive behaviors [144]. We observed decreased hippocampal DAT in young adult females compared to young adult males. Along with this sex difference, we observed that young adult female rats had decreased hippocampal DAT, NR2A, and EGR-1 compared to pubertal female rats. There are a paucity of studies investigating hippocampal DAT expression, as prior studies using Wistar, F344, and Sprague Dawley rats showed low DAT expression in the hippocampus [145–147]. Similarly, the data on sex differences in marble burying behavior is unclear. Previous marble burying studies were conducted using C57BL/6 mice, Wistar rats, and Sprague Dawley rats [148–151], which found either no sex difference [148, 149], female bias [150], or male bias in marble burying behavior [151]. Few studies on marble burying behavior incorporate both males and females [152].

## Limitations

This study has several strengths that include a broad behavior battery that allows us to examine social, cognitive, and motor functions, along with USVs. However, there are some limitations, such as the lack of multiple behavior tests to examine the same functional outcome. The behavioral battery used in this study included (1) marble burying, (2) social behavior, (3) open field- anxiety-like assessments, (4) Morris water maze, (5) USVs [9], and (6) open field-locomotor assessments [9]. These behavior tests were chosen to examine late gestation maturation domains and behavioral functions linked with USVs (e.g., motor, social, and anxiety-like functions [43–47]), while avoiding a testing battery confound [48–50]. Therefore, we are unable to broadly generalize these findings. Another limitation of this study is that we did not conduct vaginal smears for estrous cycle staging (the gold standard) to delineate if females were in high- or low-estrogen physiological states [153, 154]. We chose not to conduct vaginal smears to ensure that we treated both males and females the same and to avoid the risk of increased corticosterone and spatial memory deficits that could occur in response to vaginal lavage [155].

## Perspectives and significance

Hypoxia-associated pregnancy complications can increase the risk for chronic impairments in offspring, which are dependent on the sex and age. In response to late gestational CIH, we observed both sex- and age-dependent effects. Both pubertal male and female offspring exposed to gestational hypoxia exhibited decreased USVs [9], but the behavioral phenotype associated with this suppression in USVs was dependent on sex. In pubertal female offspring, gestational hypoxia induced social function impairments, repetitive behaviors, and corticosterone dysregulation but had no effect on cognitive function. In pubertal male offspring, gestational hypoxia induced cognitive dysfunction but had no effect on social behaviors. Some of these CIH induced behaviors were maintained into adulthood such as 1) sustained social impairments and corticosterone dysregulation in young adult females and 2) USV call production impairments in young adult males [9]. Importantly, the results from our study indicate that short-term hypoxia-associated pregnancy complications during late gestation can have long-term effects on offspring that impact social, endocrine, and cognitive maturation.

### Supplementary Information

**Additional file 1: Figure S1.** Behavioral assay depictions. Layout of marble behavior (A). Diagram of social disengagement (B).

**Additional file 2: Figure S2.** Exploratory behavior. Age increased exploratory behavior in both female offspring (A) and male offspring (B). A greater age difference was observed in gestational CIH exposed young adult males (B). Analyzed by Two-way ANOVA with Fisher’s LSD multiple comparisons tests. ANOVA significance indicated by: * = age; Post-hoc significance indicated by: # versus normoxic puberty, ### versus CIH puberty; p ≤ 0.05.

**Additional file 3: Figure S3.** Social disengagement. No effect of age was observed in female offspring (A). Gestational CIH increased social disengagement in females regardless of age (A). No effect of age or gestational CIH was observed in males (B). Analyzed by Two-way ANOVA with Fisher’s LSD multiple comparisons tests. ANOVA significance indicated by: ** = CIH; p ≤ 0.05.

**Additional file 4: Figure S4.** Repetitive behavior. Pubertal gestational CIH females had increased marble burying, while young adult gestational CIH females had decreased marble burying (A). Normoxic young adult females buried more marbles than normoxic pubertal females (A). Young adult males buried more marbles than pubertal males regardless of gestational CIH (B). Analyzed by Two-way ANOVA with Fisher’s LSD multiple comparisons tests. ANOVA significance indicated by: * = age, *** = interaction; Post-hoc significance indicated by: # versus normoxic puberty; p ≤ 0.05.

**Additional file 5: Figure S5.** Anxiety-like behavior. Young adult rats spent more time in the center of the open field compared to pubertal rats, regardless of sex or gestational CIH (A, B). Young adult rats entered the center of the open field more than pubertal rats, regardless of sex or gestational CIH (C, D). Normalized by square-root transformation (C, D). Analyzed by Two-way ANOVA with Fisher’s LSD multiple comparisons tests. ANOVA significance indicated by: * = age; Post-hoc significance indicated by: # versus normoxic puberty, ## versus normoxic young adult, ### versus CIH puberty; p ≤ 0.05.

**Additional file 6: Figure S6.** Spatial memory. No effect of age or gestational CIH on pathlength to target during Morris water maze probe trial was observed in female offspring (A). Pubertal gestational CIH males had increased pathlength to probe trial target, while young adult gestational CIH males had decreased pathlength to probe trial target (B). Analyzed by Two-way ANOVA with Fisher’s LSD multiple comparisons tests. ANOVA significance indicated by: *** = interaction; p ≤ 0.05.

**Additional file 7: Figure S7.** Puberty hippocampal protein expression. Representative images of CA1 Western blots (A-C). Representative images of dentate gyrus Western blots (D, E). Full representative blot images of NR2A (A), MAO-A (B), DAT (C), EGR-1 (D), and DC (E). Lanes 1, 5: Male Normoxic; Lanes 2, 6: Male CIH; Lanes 3, 7: Female Normoxic; Lanes 4, 8: Female CIH;* DAT: Dopamine Transporter; DC: Doublecortin; EGR-1: Early Growth Response 1; MAO-A: Monoamine Oxidase A; NR2A: NMDA Receptor 2A.*

**Additional file 8: Figure S8.** Young adult hippocampal protein expression. Representative images of CA1 Western blots (A-C). Representative images of dentate gyrus Western blots (D, E). Full representative blot images of NR2A (A), MAO-A (B), DAT (C), EGR-1 (D), and DC (E). Lanes 1, 5: Male Normoxic; Lanes 2, 6: Male CIH; Lanes 3, 7: Female Normoxic; Lanes 4, 8: Female CIH;* DAT: Dopamine Transporter; DC: Doublecortin; EGR-1: Early Growth Response 1; MAO-A: Monoamine Oxidase A; NR2A: NMDA Receptor 2A.*

**Additional file 9: Table S1.** Comparison of conspecific group sizes during social behaviors. Analyzed by Two-way ANOVA with Fisher’s LSD multiple comparisons tests. All values presented as mean ± SD. n.d. = no data for analysis.

**Additional file 10: Table S2.** Aggressive behaviors. Aggressive behavior composite score comprised of total kicking, biting, dominance posture, boxing, fighting behaviors observed. Analyzed by Two-way ANOVA with Fisher’s LSD multiple comparisons tests. All values presented as mean ± SD. n.d. = no data for analysis.

**Additional file 11: Table S3.** Morris water maze latency to target during probe trial. Latency (sec) for rats to find the target location during the probe trial. Analyzed by Two-way ANOVA with Fisher’s LSD multiple comparisons tests. All values presented as mean ± SD.

**Additional file 12: Table S4.** Comparison of gestational hypoxia parameters on social and cognitive offspring outcomes. *SD* Sprague–Dawley, *GD* gestational day.

## Data Availability

The datasets used and/or analyzed during the current study are available from the corresponding author upon reasonable request.

## References

[1] Nalivaeva NN, Turner AJ, Zhuravin IA (2018). Role of prenatal hypoxia in brain development, cognitive functions, and neurodegeneration. Front Neurosci.

[2] Qu H, Khalil RA (2020). Vascular mechanisms and molecular targets in hypertensive pregnancy and preeclampsia. Am J Physiol Heart Circ Physiol.

[3] Tarvonen M, Hovi P, Sainio S, Vuorela P, Andersson S, Teramo K (2021). Intrapartal cardiotocographic patterns and hypoxia-related perinatal outcomes in pregnancies complicated by gestational diabetes mellitus. Acta Diabetol.

[4] Lahti-Pulkkinen M, Girchenko P, Tuovinen S, Sammallahti S, Reynolds RM, Lahti J (2020). Maternal hypertensive pregnancy disorders and mental disorders in children. Hypertension.

[5] Dominguez JE, Street L, Louis J (2018). Management of obstructive sleep apnea in pregnancy. Obstet Gynecol Clin North Am.

[6] Facco FL, Parker CB, Reddy UM, Silver RM, Koch MA, Louis JM (2017). Association between sleep-disordered breathing and hypertensive disorders of pregnancy and gestational diabetes mellitus. Obstet Gynecol.

[7] Liu L, Su G, Wang S, Zhu B (2019). The prevalence of obstructive sleep apnea and its association with pregnancy-related health outcomes: a systematic review and meta-analysis. Sleep Breath.

[8] Pien GW, Pack AI, Jackson N, Maislin G, Macones GA, Schwab RJ (2014). Risk factors for sleep-disordered breathing in pregnancy. Thorax.

[9] Wilson EN, Mabry S, Bradshaw JL, Gardner JJ, Rybalchenko N, Engelland R (2022). Gestational hypoxia in late pregnancy differentially programs subcortical brain maturation in male and female rat offspring. Biol Sex Differ.

[10] Wang B, Zeng H, Liu J, Sun M (2021). Effects of prenatal hypoxia on nervous system development and related diseases. Front Neurosci.

[11] Bin YS, Cistulli PA, Roberts CL, Ford JB (2017). Childhood health and educational outcomes associated with maternal sleep apnea: a population record-linkage study. Sleep.

[12] Ream M, Ray AM, Chandra R, Chikaraishi DM (2008). Early fetal hypoxia leads to growth restriction and myocardial thinning. Am J Physiol Regul Integr Comp Physiol.

[13] Lu Q, Zhang X, Wang Y, Li J, Xu Y, Song X (2021). Sleep disturbances during pregnancy and adverse maternal and fetal outcomes: a systematic review and meta-analysis. Sleep Med Rev.

[14] Torche F, Kleinhaus K (2012). Prenatal stress, gestational age and secondary sex ratio: the sex-specific effects of exposure to a natural disaster in early pregnancy. Hum Reprod.

[15] Global Pregnancy C, Schalekamp-Timmermans S, Arends LR, Alsaker E, Chappell L, Hansson S (2017). Fetal sex-specific differences in gestational age at delivery in pre-eclampsia: a meta-analysis. Int J Epidemiol.

[16] Lorente-Pozo S, Parra-Llorca A, Torres B, Torres-Cuevas I, Nunez-Ramiro A, Cernada M (2018). Influence of sex on gestational complications, fetal-to-neonatal transition, and postnatal adaptation. Front Pediatr.

[17] Weiss SJ, Musana JW (2022). Symptoms of maternal psychological distress during pregnancy: sex-specific effects for neonatal morbidity. J Perinat Med.

[18] Gluckman PD, Hanson MA, Cooper C, Thornburg KL (2008). Effect of in utero and early-life conditions on adult health and disease. N Engl J Med.

[19] Ma RC, Tutino GE, Lillycrop KA, Hanson MA, Tam WH (2015). Maternal diabetes, gestational diabetes and the role of epigenetics in their long term effects on offspring. Prog Biophys Mol Biol.

[20] Vanderplow AM, Kermath BA, Bernhardt CR, Gums KT, Seablom EN, Radcliff AB (2022). A feature of maternal sleep apnea during gestation causes autism-relevant neuronal and behavioral phenotypes in offspring. PLoS Biol.

[21] Wang W, Tang J, Zhong M, Chen J, Li T, Dai Y (2021). HIF-1 alpha may play a role in late pregnancy hypoxia-induced autism-like behaviors in offspring rats. Behav Brain Res.

[22] Piesova M, Koprdova M, Ujhazy E, Krskova L, Olexova L, Morova M (2020). Impact of prenatal hypoxia on the development and behavior of the rat offspring. Physiol Res.

[23] Cristancho AG, Gadra EC, Samba IM, Zhao C, Ouyang M, Magnitsky S (2022). Deficits in seizure threshold and other behaviors in adult mice without gross neuroanatomic injury after late gestation transient prenatal hypoxia. Dev Neurosci.

[24] Fan JM, Wang X, Hao K, Yuan Y, Chen XQ, Du JZ (2013). Upregulation of PVN CRHR1 by gestational intermittent hypoxia selectively triggers a male-specific anxiogenic effect in rat offspring. Horm Behav.

[25] Viner RM, Allen NB, Patton GC. Puberty, Developmental Processes, and Health Interventions. In: Bundy DAP, Silva ND, Horton S, Jamison DT, Patton GC, editors. Child and Adolescent Health and Development. 3rd ed. Washington (DC)2017. doi:10.1596/978-1-4648-0423-6_ch9. 30212144

[26] Cunningham RL, Lumia AR, McGinnis MY (2013). Androgenic anabolic steroid exposure during adolescence: ramifications for brain development and behavior. Horm Behav.

[27] Yang Y, Wang JZ (2017). From structure to behavior in basolateral amygdala-hippocampus circuits. Front Neural Circuits.

[28] Banker SM, Gu X, Schiller D, Foss-Feig JH (2021). Hippocampal contributions to social and cognitive deficits in autism spectrum disorder. Trends Neurosci.

[29] Cominski TP, Jiao X, Catuzzi JE, Stewart AL, Pang KC (2014). The role of the hippocampus in avoidance learning and anxiety vulnerability. Front Behav Neurosci.

[30] Gandhi T, Lee CC (2020). Neural mechanisms underlying repetitive behaviors in rodent models of autism spectrum disorders. Front Cell Neurosci.

[31] Snyder B, Duong P, Trieu J, Cunningham RL (2018). Androgens modulate chronic intermittent hypoxia effects on brain and behavior. Horm Behav.

[32] Brockmann MD, Kukovic M, Schonfeld M, Sedlacik J, Hanganu-Opatz IL (2013). Hypoxia-ischemia disrupts directed interactions within neonatal prefrontal-hippocampal networks. PLoS ONE.

[33] Prabhakar NR, Peng YJ, Nanduri J (2020). Hypoxia-inducible factors and obstructive sleep apnea. J Clin Invest.

[34] Tamminga CA, Southcott S, Sacco C, Wagner AD, Ghose S (2012). Glutamate dysfunction in hippocampus: relevance of dentate gyrus and CA3 signaling. Schizophr Bull.

[35] Zarrindast MR, Khakpai F (2015). The modulatory role of dopamine in anxiety-like behavior. Arch Iran Med.

[36] Tian J, Stucky CS, Wang T, Muma NA, Johnson M, Du H (2023). Mitochondrial dysfunction links to impaired hippocampal serotonin release in a mouse model of Alzheimer's disease. J Alzheimers Dis.

[37] Panizzon MS, Hauger RL, Xian H, Jacobson K, Lyons MJ, Franz CE (2018). Interactive effects of testosterone and cortisol on hippocampal volume and episodic memory in middle-aged men. Psychoneuroendocrinology.

[38] Frick KM, Kim J, Koss WA (2018). Estradiol and hippocampal memory in female and male rodents. Curr Opin Behav Sci.

[39] Chamniansawat S, Sawatdiyaphanon C (2018). Age-related memory impairment associated with decreased endogenous estradiol in the hippocampus of female rats. Int J Toxicol.

[40] Tronche C, Pierard C, Coutan M, Chauveau F, Liscia P, Beracochea D (2010). Increased stress-induced intra-hippocampus corticosterone rise associated with memory impairments in middle-aged mice. Neurobiol Learn Mem.

[41] Rice D, Barone S (2000). Critical periods of vulnerability for the developing nervous system: evidence from humans and animal models. Environ Health Perspect.

[42] Bajic D, Canto Moreira N, Wikstrom J, Raininko R (2011). Development of the hippocampal region demonstrated by fetal MRI. A preliminary report. Neuroradiol J.

[43] Blanchard RJ, Blanchard DC, Agullana R, Weiss SM (1991). Twenty-two kHz alarm cries to presentation of a predator, by laboratory rats living in visible burrow systems. Physiol Behav.

[44] Portfors CV (2007). Types and functions of ultrasonic vocalizations in laboratory rats and mice. J Am Assoc Lab Anim Sci.

[45] Knutson B, Burgdorf J, Panksepp J (1998). Anticipation of play elicits high-frequency ultrasonic vocalizations in young rats. J Comp Psychol.

[46] Grant LM, Kelm-Nelson CA, Hilby BL, Blue KV, Paul Rajamanickam ES, Pultorak JD (2015). Evidence for early and progressive ultrasonic vocalization and oromotor deficits in a PINK1 gene knockout rat model of Parkinson's disease. J Neurosci Res.

[47] Berz AC, Wohr M, Schwarting RKW (2021). Response calls evoked by playback of natural 50-kHz ultrasonic vocalizations in rats. Front Behav Neurosci.

[48] Sare RM, Lemons A, Smith CB (2021). Behavior testing in rodents: highlighting potential confounds affecting variability and reproducibility. Brain Sci.

[49] McIlwain KL, Merriweather MY, Yuva-Paylor LA, Paylor R (2001). The use of behavioral test batteries: effects of training history. Physiol Behav.

[50] Blokland A, Ten Oever S, van Gorp D, van Draanen M, Schmidt T, Nguyen E (2012). The use of a test battery assessing affective behavior in rats: order effects. Behav Brain Res.

[51] McGee SR, Rose GM, Jensik PJ (2020). Impaired memory and marble burying activity in deformed epidermal autoregulatory factor 1 (Deaf1) conditional knockout mice. Behav Brain Res.

[52] Gawali NB, Chowdhury AA, Kothavade PS, Bulani VD, Nagmoti DM, Juvekar AR (2016). Involvement of nitric oxide in anticompulsive-like effect of agmatine on marble-burying behaviour in mice. Eur J Pharmacol.

[53] Felix-Ortiz AC, Tye KM (2014). Amygdala inputs to the ventral hippocampus bidirectionally modulate social behavior. J Neurosci.

[54] Cunningham RL, Lumia AR, McGinnis MY (2012). Androgen receptors, sex behavior, and aggression. Neuroendocrinology.

[55] Angoa-Perez M, Kane MJ, Briggs DI, Francescutti DM, Kuhn DM (2013). Marble burying and nestlet shredding as tests of repetitive, compulsive-like behaviors in mice. J Vis Exp.

[56] Thomas A, Burant A, Bui N, Graham D, Yuva-Paylor LA, Paylor R (2009). Marble burying reflects a repetitive and perseverative behavior more than novelty-induced anxiety. Psychopharmacology.

[57] Vanderschuren LJ, Achterberg EJ, Trezza V (2016). The neurobiology of social play and its rewarding value in rats. Neurosci Biobehav Rev.

[58] Scholl JL, Afzal A, Fox LC, Watt MJ, Forster GL (2019). Sex differences in anxiety-like behaviors in rats. Physiol Behav.

[59] Sturman O, Germain PL, Bohacek J (2018). Exploratory rearing: a context- and stress-sensitive behavior recorded in the open-field test. Stress.

[60] Cunningham RL, McGinnis MY (2007). Factors influencing aggression toward females by male rats exposed to anabolic androgenic steroids during puberty. Horm Behav.

[61] Cunningham RL, McGinnis MY (2006). Physical provocation of pubertal anabolic androgenic steroid exposed male rats elicits aggression towards females. Horm Behav.

[62] Cunningham RL, McGinnis MY (2008). Prepubertal social subjugation and anabolic androgenic steroid-induced aggression in male rats. J Neuroendocrinol.

[63] Prut L, Belzung C (2003). The open field as a paradigm to measure the effects of drugs on anxiety-like behaviors: a review. Eur J Pharmacol.

[64] Gallagher M, Burwell R, Burchinal M (2015). Severity of spatial learning impairment in aging: development of a learning index for performance in the Morris water maze. Behav Neurosci.

[65] Snyder B, Shell B, Cunningham JT, Cunningham RL (2017). Chronic intermittent hypoxia induces oxidative stress and inflammation in brain regions associated with early-stage neurodegeneration. Physiol Rep.

[66] Shell B, Farmer GE, Nedungadi TP, Wang LA, Marciante AB, Snyder B (2019). Angiotensin type 1a receptors in the median preoptic nucleus support intermittent hypoxia-induced hypertension. Am J Physiol Regul Integr Comp Physiol.

[67] Garza-Contreras J, Duong P, Snyder BD, Schreihofer DA, Cunningham RL (2017). Presence of androgen receptor variant in neuronal lipid rafts. eNeuro..

[68] Paxinos G, Watson C (1998). The rat brain in stereotaxic coordinates.

[69] Balapattabi K, Little JT, Bachelor ME, Cunningham RL, Cunningham JT (2021). Sex differences in the regulation of vasopressin and oxytocin secretion in bile duct-ligated rats. Neuroendocrinology.

[70] Wilson EN, Anderson M, Snyder B, Duong P, Trieu J, Schreihofer DA (2018). Chronic intermittent hypoxia induces hormonal and male sexual behavioral changes: hypoxia as an advancer of aging. Physiol Behav.

[71] Snyder B, Duong P, Tenkorang M, Wilson EN, Cunningham RL (2018). Rat strain and housing conditions alter oxidative stress and hormone responses to chronic intermittent hypoxia. Front Physiol.

[72] Tenkorang MAA, Duong P, Cunningham RL (2019). NADPH oxidase mediates membrane androgen receptor-induced neurodegeneration. Endocrinology.

[73] Duong P, Tenkorang MAA, Trieu J, McCuiston C, Rybalchenko N, Cunningham RL (2020). Neuroprotective and neurotoxic outcomes of androgens and estrogens in an oxidative stress environment. Biol Sex Differ.

[74] Fadeyibi O, Rybalchenko N, Mabry S, Nguyen DH, Cunningham RL (2022). The Role of Lipid Rafts and Membrane Androgen Receptors in Androgen's Neurotoxic Effects. J Endocr Soc..

[75] Tsien JZ, Huerta PT, Tonegawa S (1996). The essential role of hippocampal CA1 NMDA receptor-dependent synaptic plasticity in spatial memory. Cell.

[76] Burket JA, Benson AD, Tang AH, Deutsch SI (2015). NMDA receptor activation regulates sociability by its effect on mTOR signaling activity. Prog Neuropsychopharmacol Biol Psychiatry.

[77] McHugh PC, Buckley DA (2015). The structure and function of the dopamine transporter and its role in CNS diseases. Vitam Horm.

[78] Swant J, Wagner JJ (2006). Dopamine transporter blockade increases LTP in the CA1 region of the rat hippocampus via activation of the D3 dopamine receptor. Learn Mem.

[79] Gadow KD, Roohi J, DeVincent CJ, Hatchwell E (2008). Association of ADHD, tics, and anxiety with dopamine transporter (DAT1) genotype in autism spectrum disorder. J Child Psychol Psychiatry.

[80] Gabriel P, Mastracchio TA, Bordner K, Jeffrey R (2020). Impact of enriched environment during adolescence on adult social behavior, hippocampal synaptic density and dopamine D2 receptor expression in rats. Physiol Behav.

[81] Segal M, Markram H, Richter-Levin G (1991). Actions of norepinephrine in the rat hippocampus. Prog Brain Res.

[82] Dolen G, Darvishzadeh A, Huang KW, Malenka RC (2013). Social reward requires coordinated activity of nucleus accumbens oxytocin and serotonin. Nature.

[83] Desjardins S, Mayo W, Vallee M, Hancock D, Le Moal M, Simon H (1997). Effect of aging on the basal expression of c-Fos, c-Jun, and Egr-1 proteins in the hippocampus. Neurobiol Aging.

[84] Wang B, Guo H, Yu H, Chen Y, Xu H, Zhao G (2021). The role of the transcription factor EGR1 in cancer. Front Oncol.

[85] Duclot F, Kabbaj M (2017). The role of early growth response 1 (EGR1) in brain plasticity and neuropsychiatric disorders. Front Behav Neurosci.

[86] Ko SW, Ao HS, Mendel AG, Qiu CS, Wei F, Milbrandt J (2005). Transcription factor Egr-1 is required for long-term fear memory and anxiety. Sheng Li Xue Bao..

[87] Hueston CM, Cryan JF, Nolan YM (2017). Adolescent social isolation stress unmasks the combined effects of adolescent exercise and adult inflammation on hippocampal neurogenesis and behavior. Neuroscience.

[88] Saaltink DJ, van Zwet EW, Vreugdenhil E (2020). Doublecortin-like is implicated in adult hippocampal neurogenesis and in motivational aspects to escape from an aversive environment in male mice. eNeuro..

[89] Hill AS, Sahay A, Hen R (2015). Increasing adult hippocampal neurogenesis is sufficient to reduce anxiety and depression-like behaviors. Neuropsychopharmacology.

[90] Seo DO, Carillo MA, Chih-Hsiung Lim S, Tanaka KF, Drew MR (2015). Adult hippocampal neurogenesis modulates fear learning through associative and nonassociative mechanisms. J Neurosci.

[91] McDonald JH (2014). Handbook of Biological Statistics.

[92] Bartke A, Steele RE, Musto N, Caldwell BV (1973). Fluctuations in plasma testosterone levels in adult male rats and mice. Endocrinology.

[93] Tothova L, Celec P, Ostatnikova D, Okuliarova M, Zeman M, Hodosy J (2013). Effect of exogenous testosterone on oxidative status of the testes in adult male rats. Andrologia.

[94] Smith MS, Freeman ME, Neill JD (1975). The control of progesterone secretion during the estrous cycle and early pseudopregnancy in the rat: prolactin, gonadotropin and steroid levels associated with rescue of the corpus luteum of pseudopregnancy. Endocrinology.

[95] Goldman PR, Vogel WH (1985). Plasma estradiol and prolactin levels and their response to stress in two strains of rat with different sensitivities to 7,12-dimethylbenz[a]anthracene-induced tumors. Cancer Lett.

[96] Endlich PW, Claudio ER, da Silva Goncalves WL, Gouvea SA, Moyses MR, de Abreu GR (2013). Swimming training prevents fat deposition and decreases angiotensin II-induced coronary vasoconstriction in ovariectomized rats. Peptides.

[97] Hamdaoui L, Naifar M, Rahmouni F, Harrabi B, Ayadi F, Sahnoun Z (2018). Subchronic exposure to kalach 360 SL-induced endocrine disruption and ovary damage in female rats. Arch Physiol Biochem.

[98] Chrysostomou V, Stone J, Valter K. Differences in Photoreceptor Sensitivity to Oxygen Stress Between Long Evans and Sprague-Dawley Rats. In: Anderson RE, Hollyfield JG, LaVail MM, editors. Retinal Degenerative Diseases: Laboratory and Therapeutic Investigations. New York, NY: Springer New York; 2010. p. 473–9. doi:10.1007/978-1-4419-1399-9_54. 10.1007/978-1-4419-1399-9_54 20238049

[99] Turner KM, Burne TH (2014). Comprehensive behavioural analysis of Long Evans and Sprague-Dawley rats reveals differential effects of housing conditions on tests relevant to neuropsychiatric disorders. PLoS ONE.

[100] Meaney MJ, Aitken DH, van Berkel C, Bhatnagar S, Sapolsky RM (1988). Effect of neonatal handling on age-related impairments associated with the hippocampus. Science.

[101] Verstraeten BSE, McCreary JK, Weyers S, Metz GAS, Olson DM (2018). Prenatal two-hit stress affects maternal and offspring pregnancy outcomes and uterine gene expression in rats: match or mismatch?†. Biol Reprod.

[102] Zucchi FCR, Yao Y, Ward ID, Ilnytskyy Y, Olson DM, Benzies K (2013). Maternal stress induces epigenetic signatures of psychiatric and neurological diseases in the offspring. PLoS ONE.

[103] Semple BD, Blomgren K, Gimlin K, Ferriero DM, Noble-Haeusslein LJ (2013). Brain development in rodents and humans: identifying benchmarks of maturation and vulnerability to injury across species. Prog Neurobiol.

[104] Bayer SA, Altman J, Russo RJ, Zhang X (1993). Timetables of neurogenesis in the human brain based on experimentally determined patterns in the rat. Neurotoxicology.

[105] Chevassus-au-Louis N, Baraban SC, Gaiarsa JL, Ben-Ari Y (1999). Cortical malformations and epilepsy: new insights from animal models. Epilepsia.

[106] Kim EH, Yum MS, Lee M, Kim EJ, Shim WH, Ko TS (2017). A new rat model of epileptic spasms based on methylazoxymethanol-induced malformations of cortical development. Front Neurol.

[107] Kortheuer KH (1929). A study of development stages of the corpus striatum of the human brain.

[108] Ferriero DM (2004). Neonatal brain injury. N Engl J Med.

[109] Mandic-Maravic V, Grujicic R, Milutinovic L, Munjiza-Jovanovic A, Pejovic-Milovancevic M (2021). Dopamine in autism spectrum disorders-focus on D2/D3 partial agonists and their possible use in treatment. Front Psychiatry.

[110] Camm EJ, Cross CM, Kane AD, Tarry-Adkins JL, Ozanne SE, Giussani DA (2021). Maternal antioxidant treatment protects adult offspring against memory loss and hippocampal atrophy in a rodent model of developmental hypoxia. FASEB J.

[111] Mao M, Yang L, Jin Z, Li LX, Wang YR, Li TT (2021). Impact of intrauterine hypoxia on adolescent and adult cognitive function in rat offspring: sexual differences and the effects of spermidine intervention. Acta Pharmacol Sin.

[112] Gozal D, Reeves SR, Row BW, Neville JJ, Guo SZ, Lipton AJ (2003). Respiratory effects of gestational intermittent hypoxia in the developing rat. Am J Respir Crit Care Med.

[113] Vargas VE, Gurung S, Grant B, Hyatt K, Singleton K, Myers SM (2017). Gestational hypoxia disrupts the neonatal leptin surge and programs hyperphagia and obesity in male offspring in the Sprague-Dawley rat. PLoS ONE.

[114] Doremus-Fitzwater TL, Varlinskaya EI, Spear LP (2009). Social and non-social anxiety in adolescent and adult rats after repeated restraint. Physiol Behav.

[115] Potasiewicz A, Holuj M, Litwa E, Gzielo K, Socha L, Popik P (2020). Social dysfunction in the neurodevelopmental model of schizophrenia in male and female rats: behavioural and biochemical studies. Neuropharmacology.

[116] Lukkes JL, Engelman GH, Zelin NS, Hale MW, Lowry CA (2012). Post-weaning social isolation of female rats, anxiety-related behavior, and serotonergic systems. Brain Res.

[117] Rodrigues SM, LeDoux JE, Sapolsky RM (2009). The influence of stress hormones on fear circuitry. Annu Rev Neurosci.

[118] Choleris E, Devidze N, Kavaliers M, Pfaff DW (2008). Steroidal/neuropeptide interactions in hypothalamus and amygdala related to social anxiety. Prog Brain Res.

[119] Burgdorf J, Kroes RA, Moskal JR, Pfaus JG, Brudzynski SM, Panksepp J (2008). Ultrasonic vocalizations of rats (*Rattus norvegicus*) during mating, play, and aggression: Behavioral concomitants, relationship to reward, and self-administration of playback. J Comp Psychol.

[120] Faraday MM (2002). Rat sex and strain differences in responses to stress. Physiol Behav.

[121] Sanchis-Olle M, Sanchez-Benito L, Fuentes S, Gagliano H, Belda X, Molina P (2021). Male long-Evans rats: an outbred model of marked hypothalamic-pituitary-adrenal hyperactivity. Neurobiol Stress.

[122] Quirós Cognuck S, Reis WL, Silva M, Debarba LK, Mecawi AS, de Paula FJA (2020). Sex differences in body composition, metabolism-related hormones, and energy homeostasis during aging in Wistar rats. Physiol Rep.

[123] Handa RJ, Burgess LH, Kerr JE, O'Keefe JA (1994). Gonadal steroid hormone receptors and sex differences in the hypothalamo-pituitary-adrenal axis. Horm Behav.

[124] Bishnoi IR, Ossenkopp KP, Kavaliers M (2021). Sex and age differences in locomotor and anxiety-like behaviors in rats: from adolescence to adulthood. Dev Psychobiol.

[125] Knight P, Chellian R, Wilson R, Behnood-Rod A, Panunzio S, Bruijnzeel AW (2021). Sex differences in the elevated plus-maze test and large open field test in adult Wistar rats. Pharmacol Biochem Behav.

[126] Kulesskaya N, Voikar V (2014). Assessment of mouse anxiety-like behavior in the light-dark box and open-field arena: role of equipment and procedure. Physiol Behav.

[127] Roof RL, Stein DG (1999). Gender differences in Morris water maze performance depend on task parameters. Physiol Behav.

[128] Le AA, Lauterborn JC, Jia Y, Wang W, Cox CD, Gall CM (2022). Prepubescent female rodents have enhanced hippocampal LTP and learning relative to males, reversing in adulthood as inhibition increases. Nat Neurosci.

[129] Satterthwaite TD, Vandekar S, Wolf DH, Ruparel K, Roalf DR, Jackson C (2014). Sex differences in the effect of puberty on hippocampal morphology. J Am Acad Child Adolesc Psychiatry.

[130] Perrot-Sinal TS, Kostenuik MA, Ossenkopp KP, Kavaliers M (1996). Sex differences in performance in the Morris water maze and the effects of initial nonstationary hidden platform training. Behav Neurosci.

[131] Qi X, Zhang K, Xu T, Yamaki VN, Wei Z, Huang M (2016). Sex differences in long-term potentiation at temporoammonic-CA1 synapses: potential implications for memory consolidation. PLoS ONE.

[132] DiCarlo GE, Wallace MT (2022). Modeling dopamine dysfunction in autism spectrum disorder: from invertebrates to vertebrates. Neurosci Biobehav Rev.

[133] Deutsch SI, Luyo ZNM, Burket JA (2022). Targeted NMDA receptor interventions for autism: developmentally determined expression of GluN2B and GluN2A-containing receptors and balanced allosteric modulatory approaches. Biomolecules.

[134] Verma D, Chakraborti B, Karmakar A, Bandyopadhyay T, Singh AS, Sinha S (2014). Sexual dimorphic effect in the genetic association of monoamine oxidase A (MAOA) markers with autism spectrum disorder. Prog Neuropsychopharmacol Biol Psychiatry.

[135] Gu F, Chauhan V, Chauhan A (2017). Monoamine oxidase-A and B activities in the cerebellum and frontal cortex of children and young adults with autism. J Neurosci Res.

[136] Choi JH, Sim SE, Kim JI, Choi DI, Oh J, Ye S (2018). Interregional synaptic maps among engram cells underlie memory formation. Science.

[137] Hainmueller T, Bartos M (2020). Dentate gyrus circuits for encoding, retrieval and discrimination of episodic memories. Nat Rev Neurosci.

[138] Simpkins KL, Guttmann RP, Dong Y, Chen Z, Sokol S, Neumar RW (2003). Selective activation induced cleavage of the NR2B subunit by calpain. J Neurosci.

[139] Dong YN, Waxman EA, Lynch DR (2004). Interactions of postsynaptic density-95 and the NMDA receptor 2 subunit control calpain-mediated cleavage of the NMDA receptor. J Neurosci.

[140] Mohammadkhani R, Ghahremani R, Salehi I, Safari S, Karimi SA, Zarei M (2022). Impairment in social interaction and hippocampal long-term potentiation at perforant pathway-dentate gyrus synapses in a prenatal valproic acid-induced rat model of autism. Brain Communications.

[141] Kinjo T, Ito M, Seki T, Fukuhara T, Bolati K, Arai H (2019). Prenatal exposure to valproic acid is associated with altered neurocognitive function and neurogenesis in the dentate gyrus of male offspring rats. Brain Res.

[142] Lee L-T, Tsai HC, Chi MH, Chang WH, Chen KC, Lee IH (2015). Lower availability of striatal dopamine transporter in generalized anxiety disorder: a preliminary two-ligand SPECT study. Int Clin Psychopharmacol.

[143] Babapoor-Farrokhran S, Zarrindast M-R, Rezayof A (2008). The dopaminergic system of ventral hippocampus is involved in the anxiety related behavior. Ann General Psychiatry.

[144] Festucci F, Annunzi E, Pepe M, Curcio G, D'Addario C, Adriani W (2022). Dopamine-transporter heterozygous rats carrying maternal wild-type allele are more vulnerable to the development of compulsive behavior. Synapse.

[145] Borgkvist A, Malmlöf T, Feltmann K, Lindskog M, Schilström B (2012). Dopamine in the hippocampus is cleared by the norepinephrine transporter. Int J Neuropsychopharmacol.

[146] Mennicken F, Savasta M, Peretti-Renucci R, Feuerstein C (1992). Autoradiographic localization of dopamine uptake sites in the rat brain with 3H-GBR 12935. J Neural Transm Gen Sect.

[147] Smolders I, Clinckers R, Meurs A, De Bundel D, Portelli J, Ebinger G (2008). Direct enhancement of hippocampal dopamine or serotonin levels as a pharmacodynamic measure of combined antidepressant-anticonvulsant action. Neuropharmacology.

[148] Witchey SK, Al Samara L, Horman BM, Stapleton HM, Patisaul HB (2020). Perinatal exposure to FireMaster(R) 550 (FM550), brominated or organophosphate flame retardants produces sex and compound specific effects on adult Wistar rat socioemotional behavior. Horm Behav.

[149] Taylor GT, Lerch S, Chourbaji S (2017). Marble burying as compulsive behaviors in male and female mice. Acta Neurobiol Exp (Wars)..

[150] Freund N, Thompson BS, Norman KJ, Einhorn P, Andersen SL (2015). Developmental emergence of an obsessive-compulsive phenotype and binge behavior in rats. Psychopharmacology.

[151] Mitra S, Bastos CP, Chesworth S, Frye C, Bult-Ito A (2017). Strain and sex based characterization of behavioral expressions in non-induced compulsive-like mice. Physiol Behav.

[152] de Brouwer G, Fick A, Harvey BH, Wolmarans DW (2019). A critical inquiry into marble-burying as a preclinical screening paradigm of relevance for anxiety and obsessive–compulsive disorder: mapping the way forward. Cogn Affect Behav Neurosci.

[153] Cora MC, Kooistra L, Travlos G (2015). Vaginal cytology of the laboratory rat and mouse: review and criteria for the staging of the estrous cycle using stained vaginal smears. Toxicol Pathol.

[154] Krentzel AA, Meitzen J (2018). Biological sex, estradiol and striatal medium spiny neuron physiology: a mini-review. Front Cell Neurosci.

[155] Becegato M, Meurer YSR, Paiva-Santos MA, Lima AC, Marinho GF, Bioni VS (2021). Impaired discriminative avoidance and increased plasma corticosterone levels induced by vaginal lavage procedure in rats. Physiol Behav.

