# Supplementary figures and images for: Sex and age differences in social and cognitive function in offspring exposed to late gestational hypoxia
Source: Biol Sex Differ. 2023 Nov 11;14:81. doi: 10.1186/s13293-023-00557-0 (PMC10640736; doi:10.1186/s13293-023-00557-0)

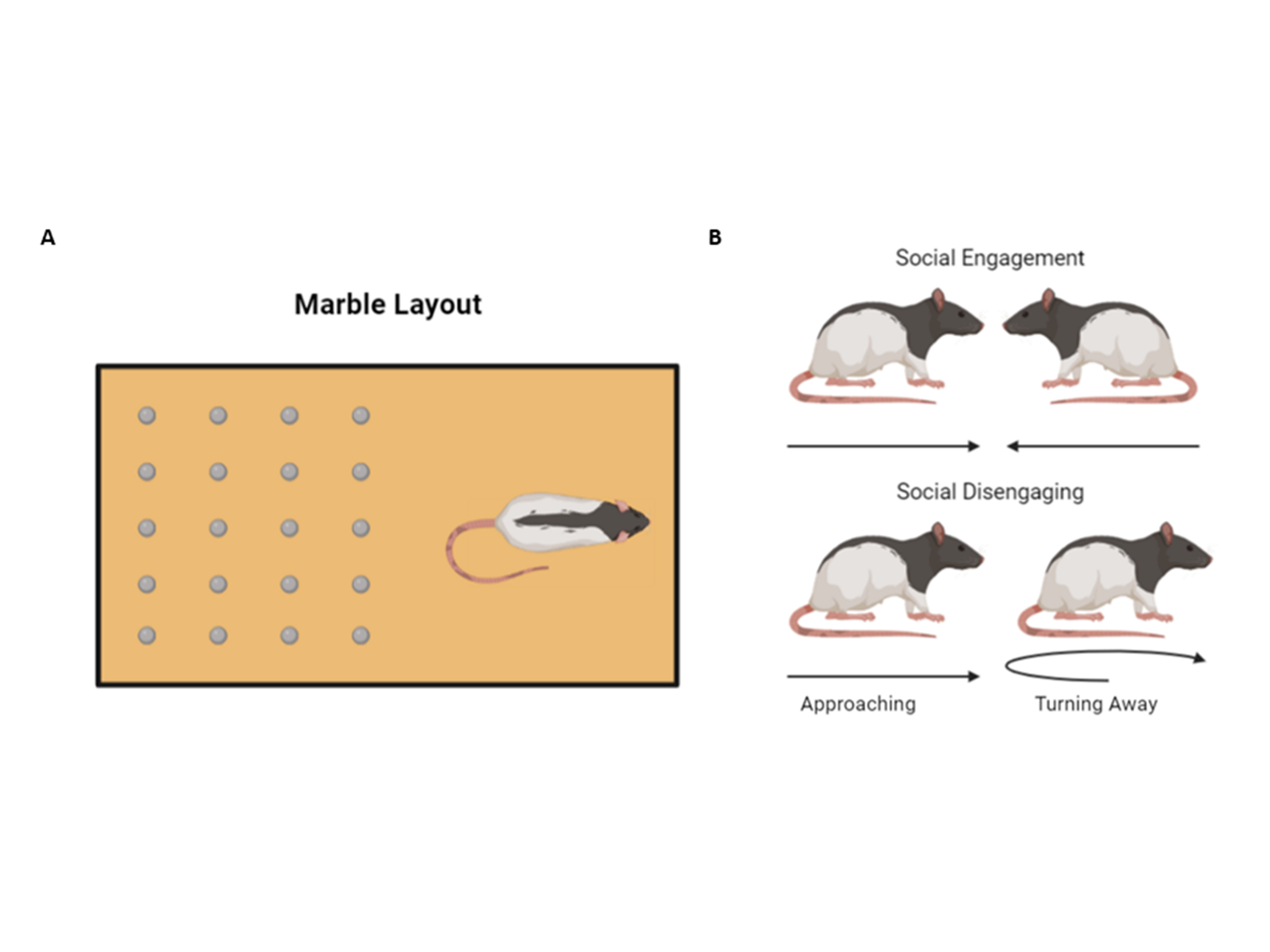

Supplement: Supplementary file 1 — Additional file 1: Figure S1. Behavioral assay depictions. Layout of marble behavior (A). Diagram of social disengagement (B). [file 13293_2023_557_MOESM1_ESM.tif]

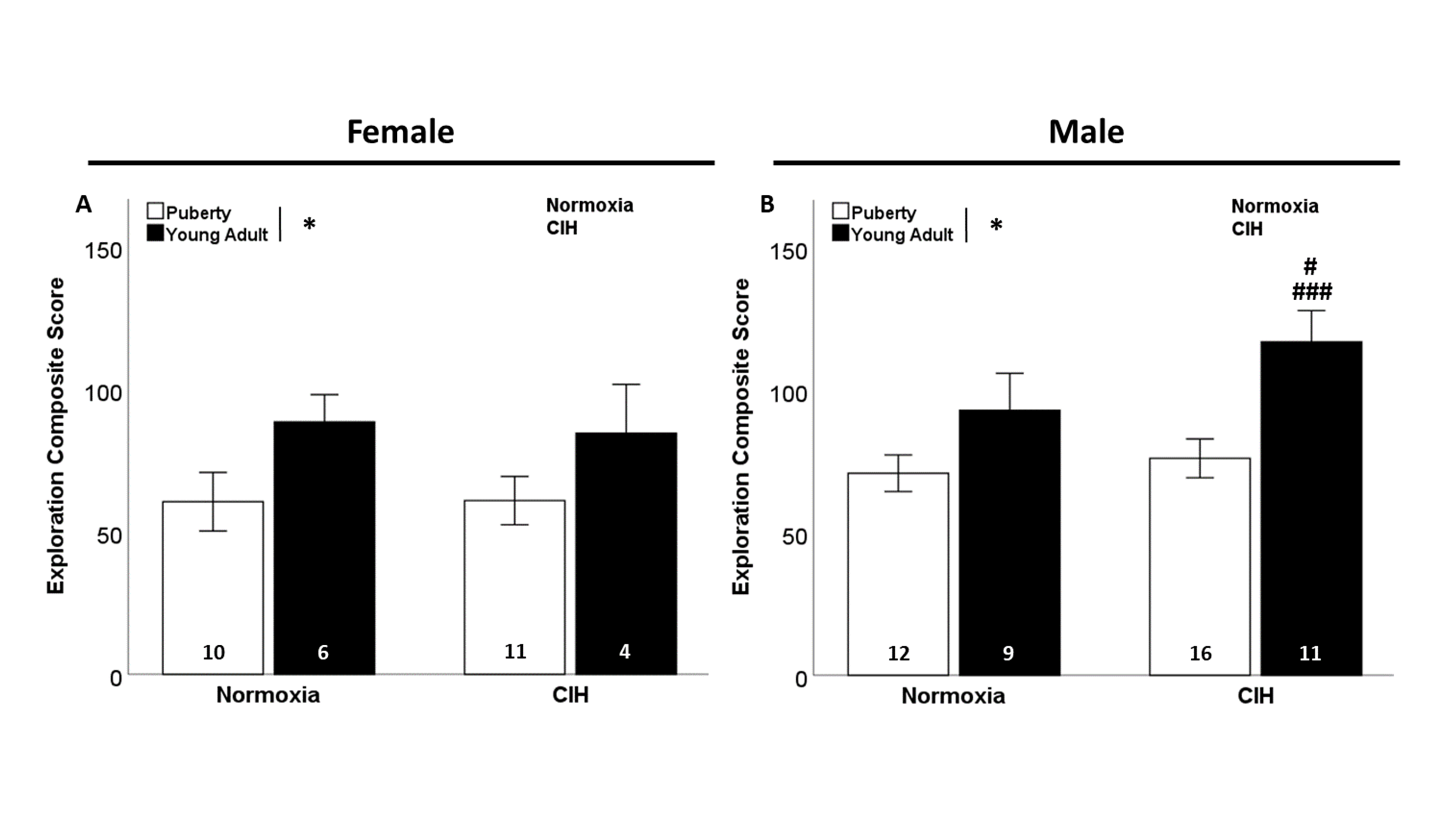

Supplement: Supplementary file 2 — Additional file 2: Figure S2. Exploratory behavior. Age increased exploratory behavior in both female offspring (A) and male offspring (B). A greater age difference was observed in gestational CIH exposed young adult males (B). Analyzed by Two-way ANOVA with Fisher’s LSD multiple comparisons tests. ANOVA significance indicated by: * = age; Post-hoc significance indicated by: # versus normoxic puberty, ### versus CIH puberty; p ≤ 0.05. [file 13293_2023_557_MOESM2_ESM.tif]

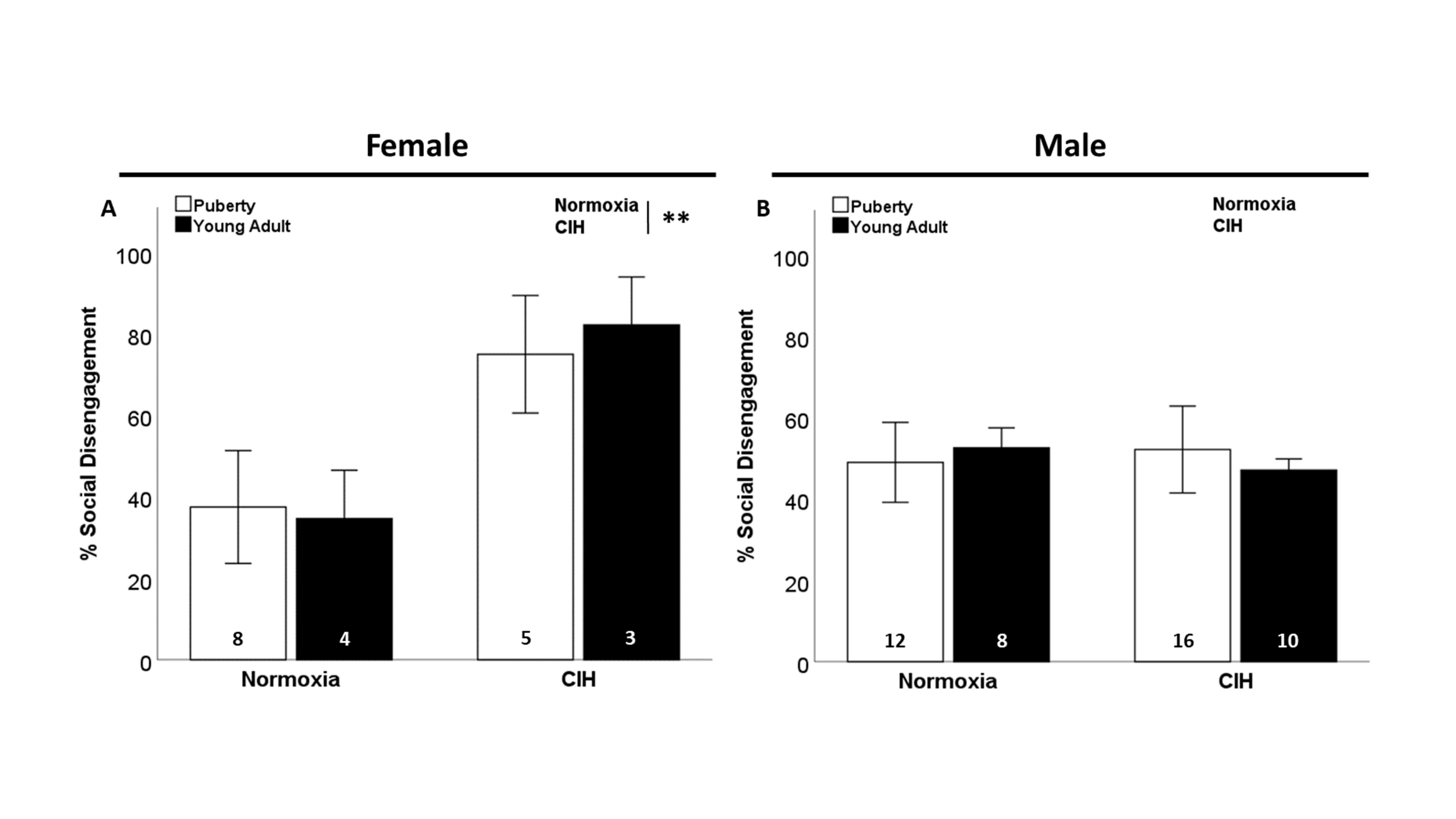

Supplement: Supplementary file 3 — Additional file 3: Figure S3. Social disengagement. No effect of age was observed in female offspring (A). Gestational CIH increased social disengagement in females regardless of age (A). No effect of age or gestational CIH was observed in males (B). Analyzed by Two-way ANOVA with Fisher’s LSD multiple comparisons tests. ANOVA significance indicated by: ** = CIH; p ≤ 0.05. [file 13293_2023_557_MOESM3_ESM.tif]

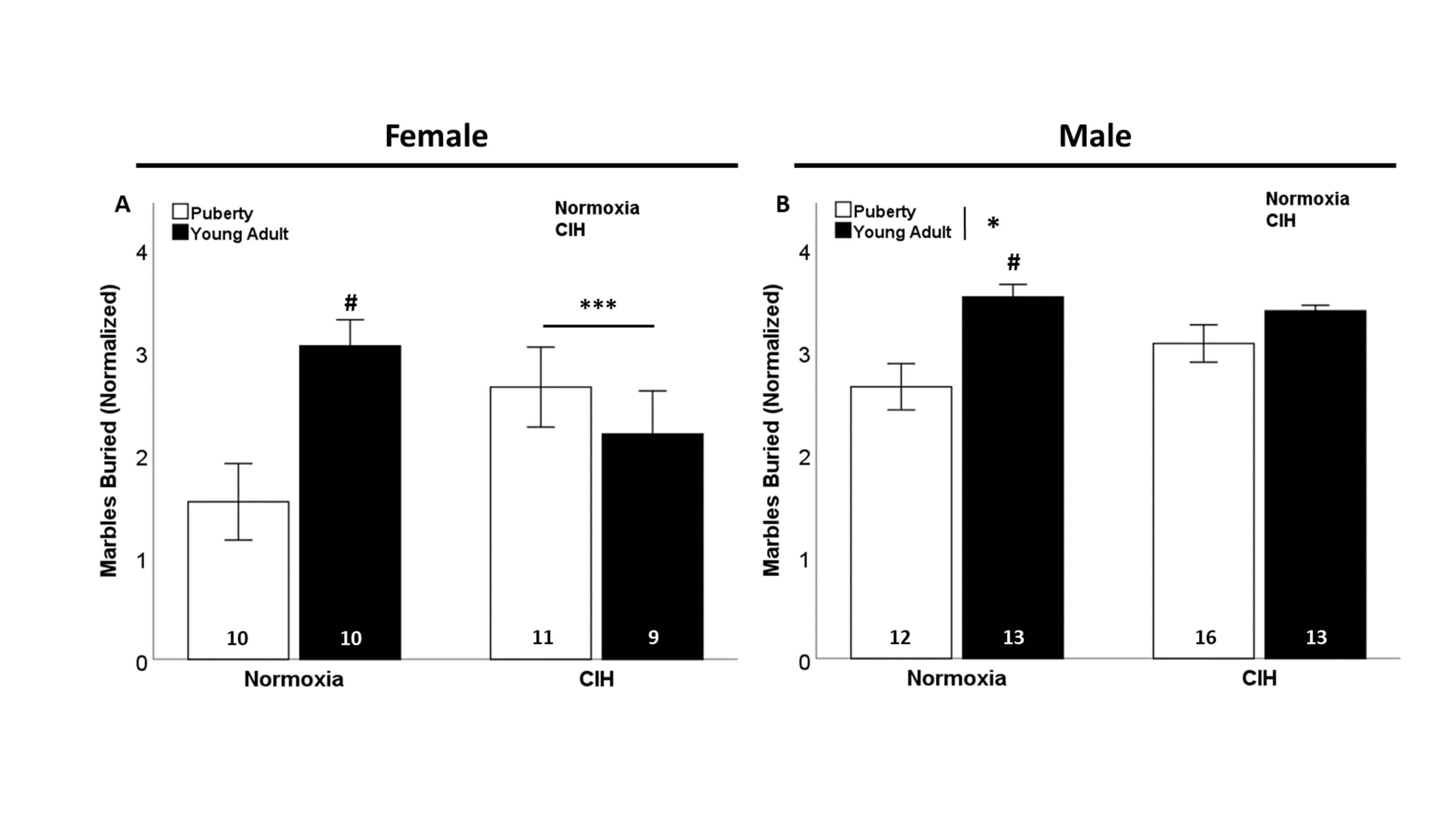

Supplement: Supplementary file 4 — Additional file 4: Figure S4. Repetitive behavior. Pubertal gestational CIH females had increased marble burying, while young adult gestational CIH females had decreased marble burying (A). Normoxic young adult females buried more marbles than normoxic pubertal females (A). Young adult males buried more marbles than pubertal males regardless of gestational CIH (B). Analyzed by Two-way ANOVA with Fisher’s LSD multiple comparisons tests. ANOVA significance indicated by: * = age, *** = interaction; Post-hoc significance indicated by: # versus normoxic puberty; p ≤ 0.05. [file 13293_2023_557_MOESM4_ESM.tif]

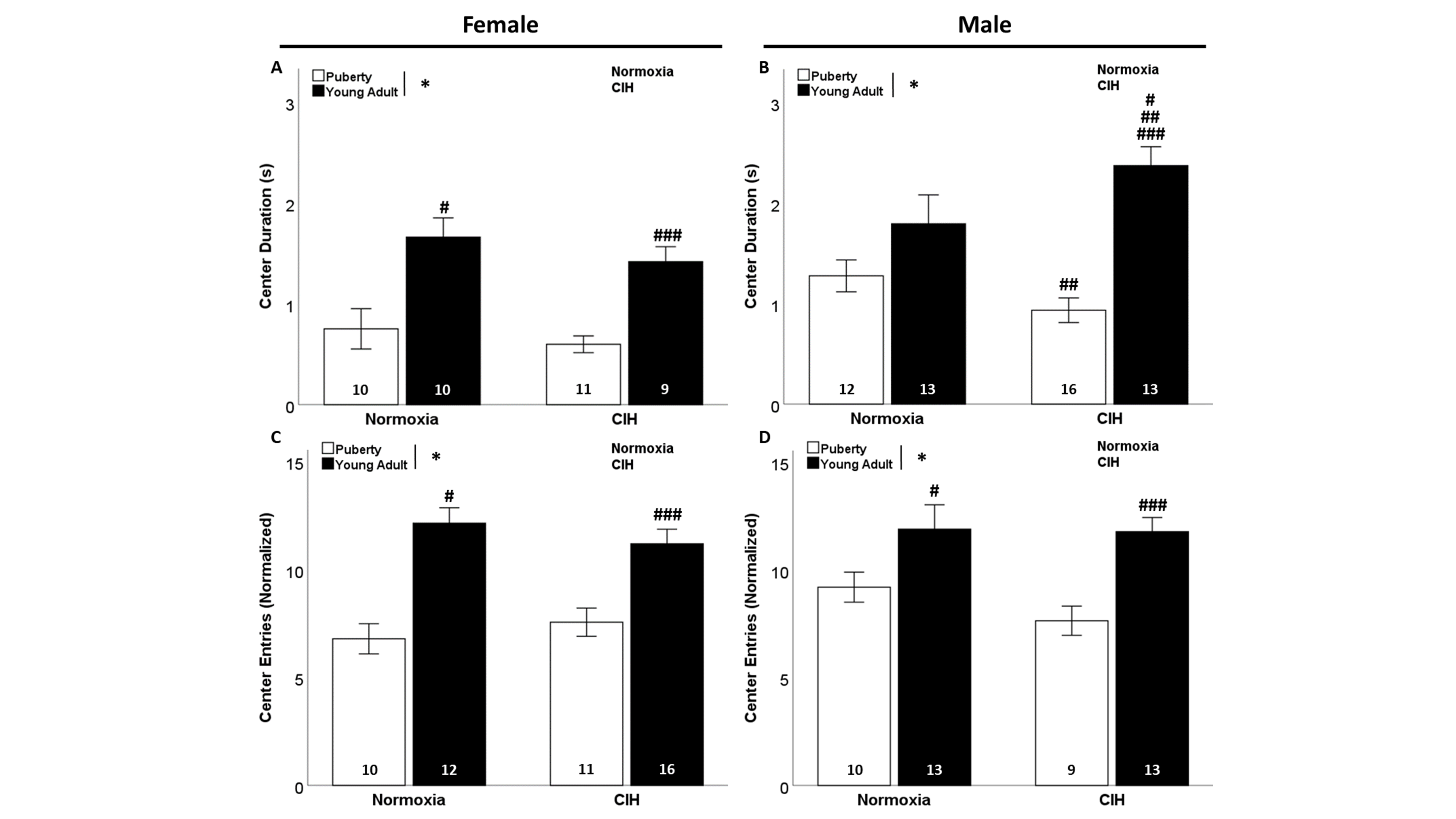

Supplement: Supplementary file 5 — Additional file 5: Figure S5. Anxiety-like behavior. Young adult rats spent more time in the center of the open field compared to pubertal rats, regardless of sex or gestational CIH (A, B). Young adult rats entered the center of the open field more than pubertal rats, regardless of sex or gestational CIH (C, D). Normalized by square-root transformation (C, D). Analyzed by Two-way ANOVA with Fisher’s LSD multiple comparisons tests. ANOVA significance indicated by: * = age; Post-hoc significance indicated by: # versus normoxic puberty, ## versus normoxic young adult, ### versus CIH puberty; p ≤ 0.05. [file 13293_2023_557_MOESM5_ESM.tif]

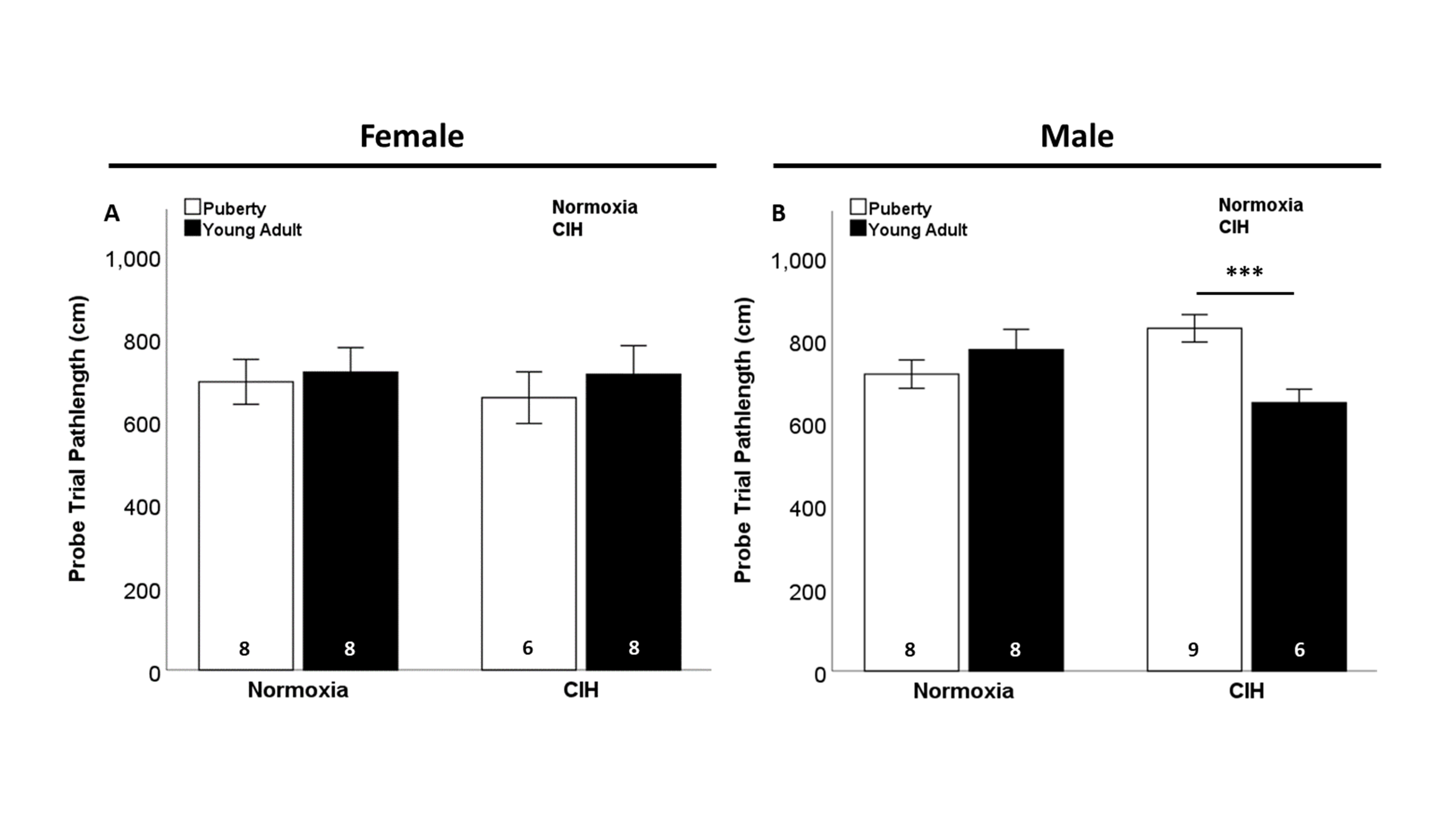

Supplement: Supplementary file 6 — Additional file 6: Figure S6. Spatial memory. No effect of age or gestational CIH on pathlength to target during Morris water maze probe trial was observed in female offspring (A). Pubertal gestational CIH males had increased pathlength to probe trial target, while young adult gestational CIH males had decreased pathlength to probe trial target (B). Analyzed by Two-way ANOVA with Fisher’s LSD multiple comparisons tests. ANOVA significance indicated by: *** = interaction; p ≤ 0.05. [file 13293_2023_557_MOESM6_ESM.tif]

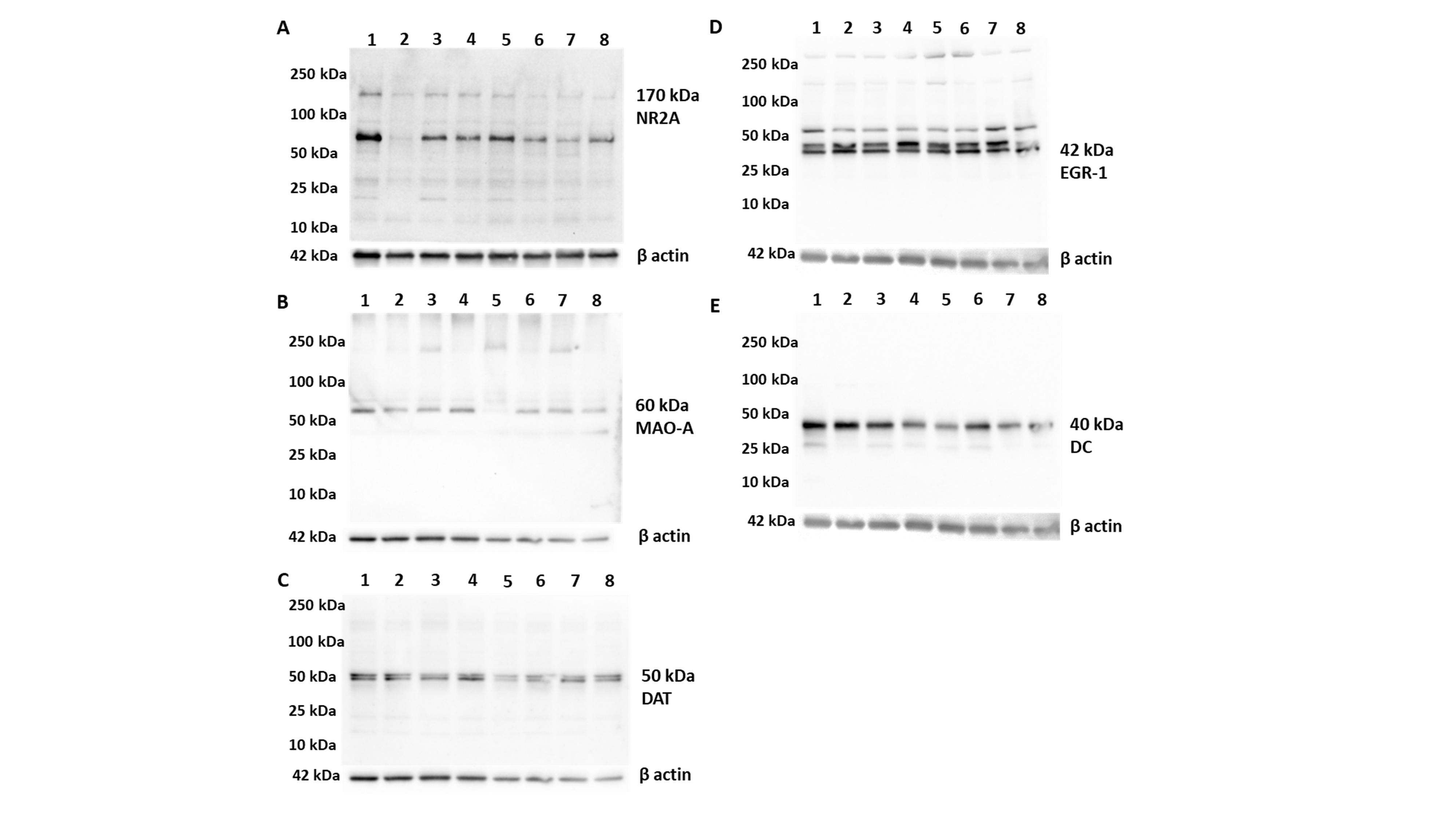

Supplement: Supplementary file 7 — Additional file 7: Figure S7. Puberty hippocampal protein expression. Representative images of CA1 Western blots (A-C). Representative images of dentate gyrus Western blots (D, E). Full representative blot images of NR2A (A), MAO-A (B), DAT (C), EGR-1 (D), and DC (E). Lanes 1, 5: Male Normoxic; Lanes 2, 6: Male CIH; Lanes 3, 7: Female Normoxic; Lanes 4, 8: Female CIH; DAT: Dopamine Transporter; DC: Doublecortin; EGR-1: Early Growth Response 1; MAO-A: Monoamine Oxidase A; NR2A: NMDA Receptor 2A. [file 13293_2023_557_MOESM7_ESM.tif]

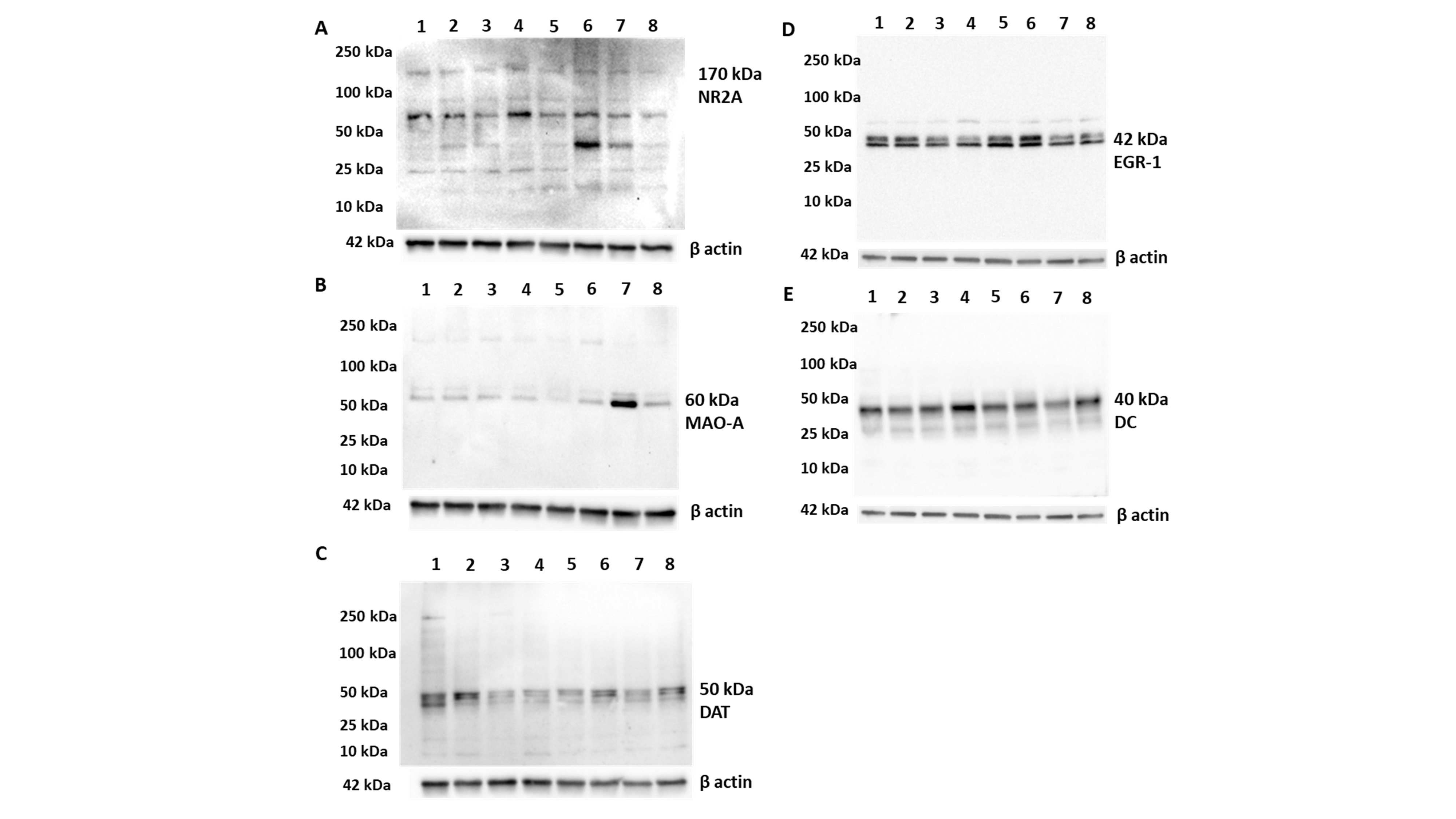

Supplement: Supplementary file 8 — Additional file 8: Figure S8. Young adult hippocampal protein expression. Representative images of CA1 Western blots (A-C). Representative images of dentate gyrus Western blots (D, E). Full representative blot images of NR2A (A), MAO-A (B), DAT (C), EGR-1 (D), and DC (E). Lanes 1, 5: Male Normoxic; Lanes 2, 6: Male CIH; Lanes 3, 7: Female Normoxic; Lanes 4, 8: Female CIH; DAT: Dopamine Transporter; DC: Doublecortin; EGR-1: Early Growth Response 1; MAO-A: Monoamine Oxidase A; NR2A: NMDA Receptor 2A. [file 13293_2023_557_MOESM8_ESM.tif]

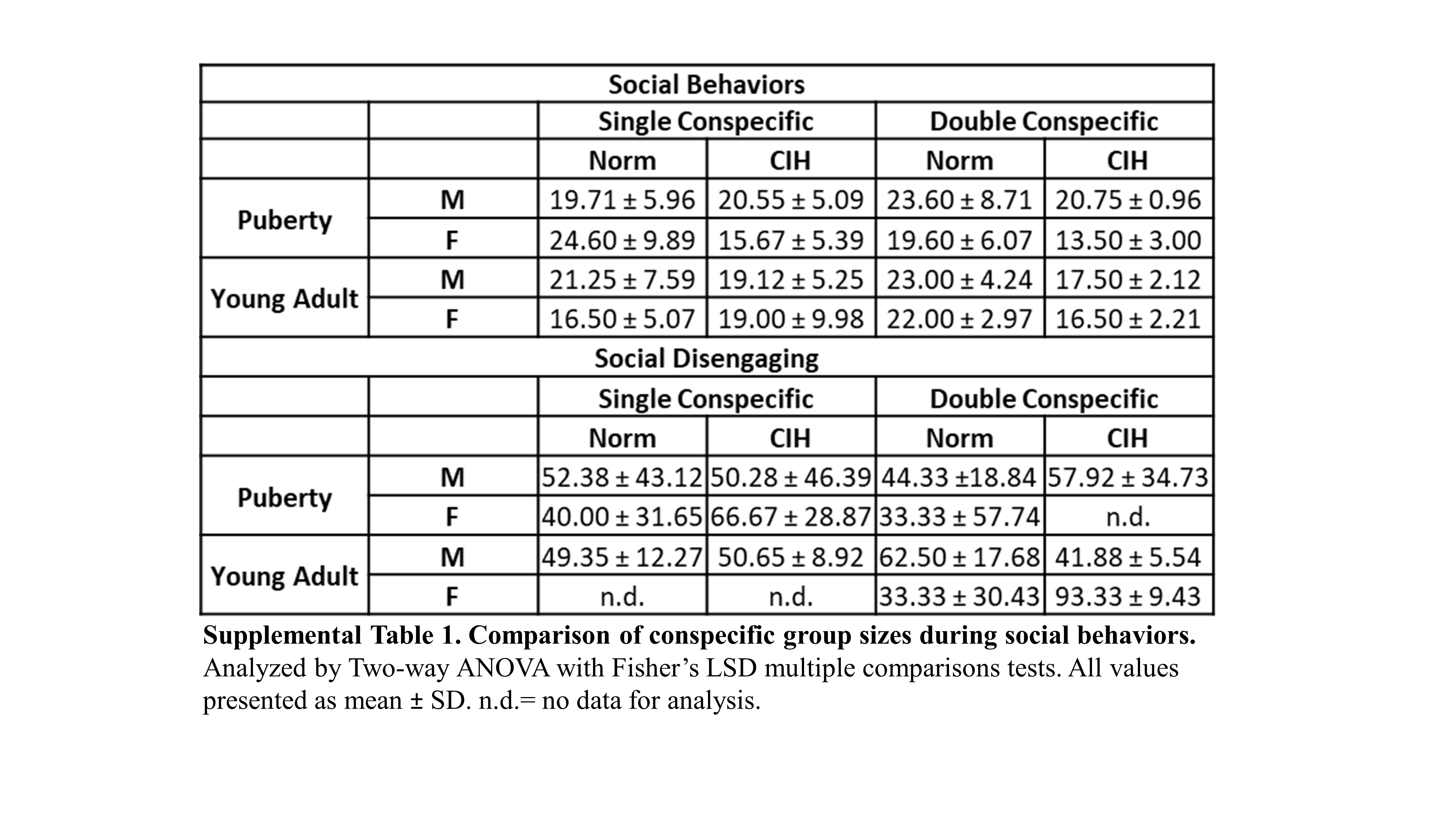

Supplement: Supplementary file 9 — Additional file 9: Table S1. Comparison of conspecific group sizes during social behaviors. Analyzed by Two-way ANOVA with Fisher’s LSD multiple comparisons tests. All values presented as mean ± SD. n.d. = no data for analysis. [file 13293_2023_557_MOESM9_ESM.tif]

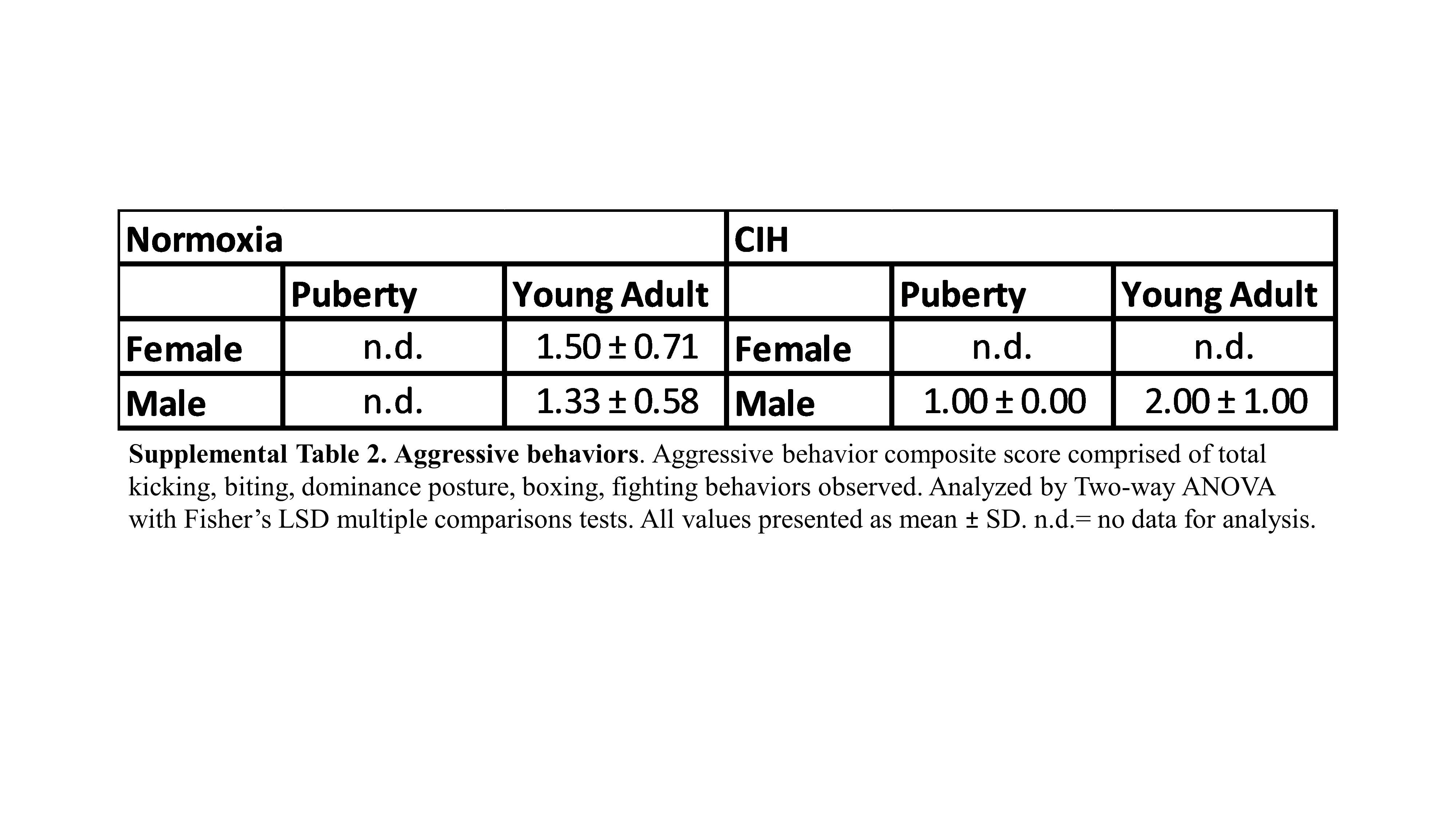

Supplement: Supplementary file 10 — Additional file 10: Table S2. Aggressive behaviors. Aggressive behavior composite score comprised of total kicking, biting, dominance posture, boxing, fighting behaviors observed. Analyzed by Two-way ANOVA with Fisher’s LSD multiple comparisons tests. All values presented as mean ± SD. n.d. = no data for analysis. [file 13293_2023_557_MOESM10_ESM.tif]

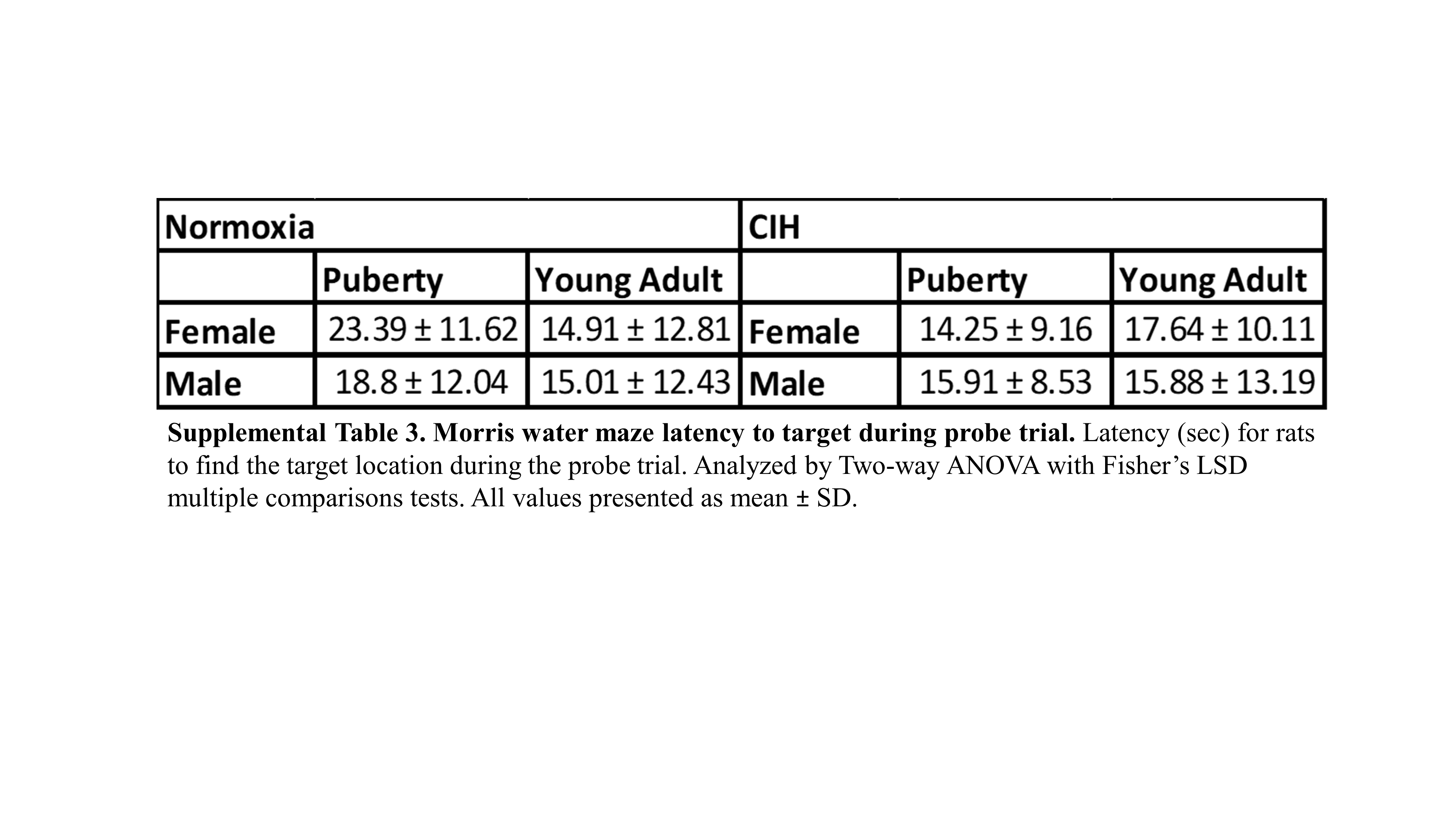

Supplement: Supplementary file 11 — Additional file 11: Table S3. Morris water maze latency to target during probe trial. Latency (sec) for rats to find the target location during the probe trial. Analyzed by Two-way ANOVA with Fisher’s LSD multiple comparisons tests. All values presented as mean ± SD. [file 13293_2023_557_MOESM11_ESM.tif]
